# Epigenetic Heterogeneity of B-Cell Lymphoma: Chromatin Modifiers

**DOI:** 10.3390/genes6041076

**Published:** 2015-10-21

**Authors:** Lydia Hopp, Lilit Nersisyan, Henry Löffler-Wirth, Arsen Arakelyan, Hans Binder

**Affiliations:** 1 Interdisciplinary Centre for Bioinformatics, Universität Leipzig, Härtelstr. 16–18, Leipzig 04107, Germany; E-Mail: wirth@izbi.uni-leipzig.de; 2 Group of Bioinformatics, Institute of Molecular Biology NAS RA, 7 Hasratyan St, Yerevan 0014, Armenia; E-Mails: l_nersisyan@mb.sci.am (L.N.); aarakelyan@sci.am (A.A.); 3 College of Science and Engineering, American University of Armenia, 40 Baghramyan Ave, Yerevan 0019, Armenia

**Keywords:** writers and erasers of epigenetic marks, methylation of histone-lysine side chains, regulation of gene expression, coupling between energy metabolism and epigenetics, plasticity of cell function, B cell maturation

## Abstract

We systematically studied the expression of more than fifty histone and DNA (de)methylating enzymes in lymphoma and healthy controls. As a main result, we found that the expression levels of nearly all enzymes become markedly disturbed in lymphoma, suggesting deregulation of large parts of the epigenetic machinery. We discuss the effect of DNA promoter methylation and of transcriptional activity in the context of mutated epigenetic modifiers such as EZH2 and MLL2. As another mechanism, we studied the coupling between the energy metabolism and epigenetics via metabolites that act as cofactors of JmjC-type demethylases. Our study results suggest that Burkitt’s lymphoma and diffuse large B-cell Lymphoma differ by an imbalance of repressive and poised promoters, which is governed predominantly by the activity of methyltransferases and the underrepresentation of demethylases in this regulation. The data further suggest that coupling of epigenetics with the energy metabolism can also be an important factor in lymphomagenesis in the absence of direct mutations of genes in metabolic pathways. Understanding of epigenetic deregulation in lymphoma and possibly in cancers in general must go beyond simple schemes using only a few modes of regulation.

## 1. Introduction

**Figure 1:**
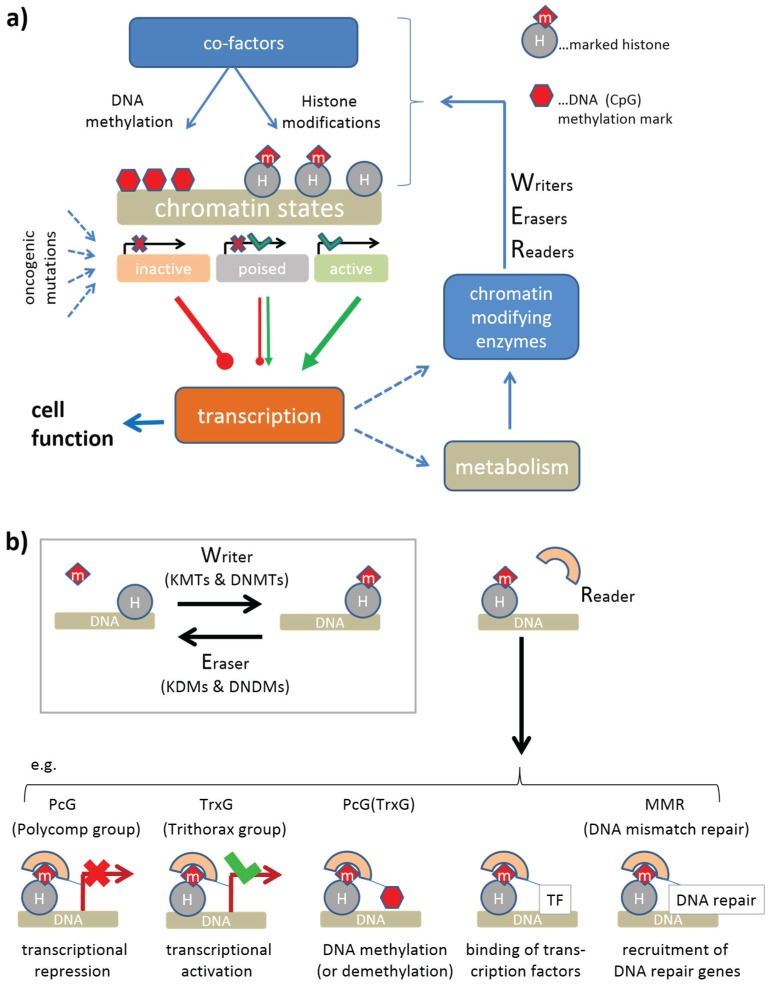
(**a**) Circuit of epigenetic regulation by chromatin modifiers: Different chromatin states are induced by a battery of histone modification, which in concert with DNA methylation, modulate transcription of the affected genes. The activity of the genes also affects the production (via transcription, translation and also posttranslational changes) of chromatin modifying enzymes, which, together with cofactors modulate the formation of different chromatin states. This feedback loop is further modulated by metabolites, which serve as cofactors. Oncogenic mutations can dis-balance this network giving rise to malignant cellular states. (**b**) Chromatin modifying enzymes comprise writers, erasers and readers of epigenetic marks at the histones and/or the DNA. Writers, such as histone (KMTs) or DNA methyltransferases (DNMTs), and erasers, such as histone (KDMs) or DNA demethylases (DNDMs), add or remove these marks, respectively. Readers recognize such marks and induce specific molecular “actions” (activation or repression of gene expression, writing or erasing of DNA or histone marks or recruitment of transcription factors or of DNA-repair genes).

The genetic changes leading to the development of human cancer are accompanied (or even driven) by alterations in the structure and modification status of chromatin, which represent an important regulatory mechanism for gene expression and genome stability [1,2]. Figure 1a provides a schematic overview of central ingredients of this mechanism: chromatin states such as euchromatin and heterochromatin, and also the more subtle activity states of gene promoters represent essential determinants of gene transcription shaping cell function and the production of chromatin modifying enzymes. These enzymes model the chromatin states via writing and erasing of epigenetic marks attached, e.g., to histone amino acid side chains such as lysines or arginines. Such marks are then read by the chromatin (re-)modeling machinery, which potentially leads to changes of chromatin structure with possible consequences for gene expression (see Figure 1b). This regulatory circuit plays not only an important role in cancer, but also in differentiation and development by switching cell activity between different regulatory programs. The activity of chromatin modifying enzymes within this regulatory circuit represents one important determinant of epigenetic regulation.

Mutations of genes coding epigenetic modifiers are initiating events in cancer that can induce an “avalanche” of downstream epigenetic effects. They can start with the aberrant expression of chromatin modifying enzymes, which leads to aberrant epigenetic marks and in consequence to aberrant chromatin states and finally to aberrant cellular activities. Mutations not directly targeting epigenetic modifiers can also induce analogous “avalanches” of epigenetic deregulation, if, for example, they hit transcription factors, which downstream regulate the expression of epigenetic modifiers. De-regulation of the epigenetic machinery can also be mediated by the metabolome, namely if mutations of genes encoding metabolic enzymes modify metabolites acting as inhibitors or activators of epigenetic enzymes. For example, mutations of the gene, which codes for isocitrate dehydrogenase 1 (IDH1) disturb the DNA methylation machinery and induce special types of brain cancer by alterations of the activity of epigenetic enzymes [3]. Perturbations of chromatin-modifying mechanisms are among the central oncogenic pathways inducing human cancer [4].

Mutations affecting epigenetic and transcriptional modifiers are also frequently found in B-cell lymphomas [2,5]. Large-scale disruptions of DNA methylation and histone modification patterns are emerging hallmarks of these diseases. B-cell lymphomas represent a very heterogeneous cancer entity due to its complex cell of origin background. It is characterized by heterogeneous DNA methylation and gene expression patterns, which strongly vary between different lymphoma subtypes [6,7,8]. These patterns also indicate profound chromatin remodeling between the cancer subtypes and also between different stages of B cell differentiation [6]. In this publication, we studied the transcriptional activity of more than fifty epigenetic modifiers in different lymphoma subtypes and healthy controls. We ask how the expression landscape of this disease is modulated by these enzymes. We discuss different modes of epigenetic regulation and review existing knowledge about selected modifiers in the context of B cell and lymphoma biology.

For a holistic view on the complex expression data landscape we make use of a machine learning method. It translates expression profiles of tens of thousands of genes into a two-dimensional map. This expression “cartography” is well suited to disentangle the heterogeneity of expression landscapes in large cancer cohorts [9,10,11]. We demonstrate how the cartography of epigenetic modifiers helps to interpret their behavior in terms of factors that mediate writing, erasing and/or reading of epigenetic marks and how they contribute to cancer genesis and progression.

## 2. Data and Methods

### 2.1. Lymphoma Gene Expression Data

Publically available gene expression data of 632 samples were taken as CEL files from Gene Expression Omnibus (GEO accession numbers GSE4475, GSE10172, GSE22470, GSE48184, GSE43677). All samples were measured in the MMML (Molecular mechanisms of malignant lymphoma) cohort [7]. Lymphoma samples were classified into five molecular subtypes as described in [10,12]: molecular (mBL, *n =* 62 samples), non-molecular Burkitt’s (non-mBL, *n =* 204), intermediate lymphoma (IntL, *n =* 255), follicular lymphoma (FL, *n =* 3) and B cell like lymphoma (BCL, *n =* 36). According to patho-histological diagnosis the molecular subtypes refer predominantly to Burkitt’s lymphoma (BL, mBL), diffuse large B-cell lymphoma (DLBCL, non-mBL) and multiple myeloma (MM, BCL). For the sake of convenience, we will assign the subtypes in the paper by their histological assignment, which will provide sufficient resolution for the purposes of this presentation. The cohort also contains healthy (naïve) B cells (17) and germinal center B (GCB, 13) cells, a lymphoma cancer cell line (32) and non-neoplastic tonsils (10) as controls [13]. The microarray expression data (Affymetrix HG-U133a) were processed as described previously [10].

### 2.2. High-Dimensional Data Portraying

**Figure 2:**
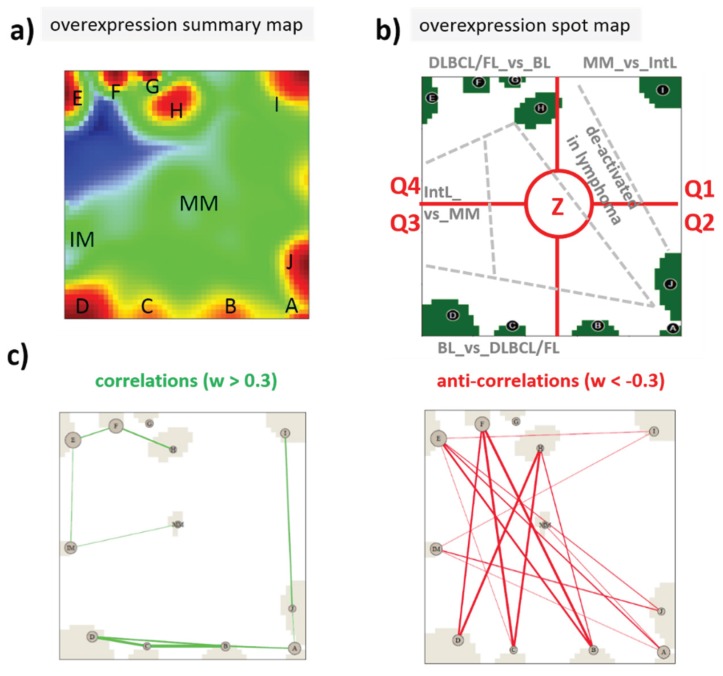
Expression SOM characteristics of lymphoma: (**a**) The overexpression spot summary map shows all overexpression spots in red which were detected in the lymphoma cohort studied [12]; (**b**) The spot map selects defined spot areas (A–J, IM and MM) representing clusters of co-expressed genes and overexpressed in a certain sample class. Accordingly, the map can be segmented into areas of characteristic differential expression between the lymphoma classes and healthy controls (e.g., BL_*vs.*_DLBCL means that the area contains genes overexpressed in BL compared with DLBCL). The dashed borderlines between these areas serve as guide for the eye inspection only. In reality, the areas are fuzzy without clear-cut borderlines. For the sake of a simple and clear description, we divide the map into four quadrants (Q1–Q4) and a central region (Z). Q1 can be assigned to genes upregulated in MM (MM_up), Q2 contains diverse deregulation patterns, Q3 can be assigned to genes specifically upregulated in BL (BL_up), Q4 is assigned to DLBCL_up and FL_up, and, finally, Z includes genes of almost invariant expression. (**c**) The correlation and anti-correlation maps show strongest correlations and anti-correlations between the spot clusters calculated by means of weighted topological overlap measures as described in [11]. They support the segmentation shown in (b). Strongly correlated spots merge within the same area, e.g., Q4 and Q3. Anticorrelated spots are found in opposite areas of the map, e.g., Q4 anti-correlates with Q2–Q3.

We used self-organizing map (SOM) machine learning as implemented in the program “oposSOM” [14] to analyze centralized log-expression data (Differential expression SOM, DexSOM) as described in [6]. All expression data were given in log_10_-scale, hence, the fold change between expression values of 0 and 1 is ten in linear scale. The DexSOM of the lymphoma classes and controls considered is described previously [6,12]. The SOM portraying method transforms the multitude of different profiles inherent in a multidimensional dataset into a two-dimensional map. A profile is defined as the series of expression values of a selected gene in all samples studied. The SOM algorithm clusters similar profiles of co-expressed genes, which in consequence occupy localized areas of the map. The basic rule of thumb to interpret SOM structure states that “the more similar two genes behave, the closer they are located in the map”. Hence, the data map obtained can be simply “read” by visual inspection revealing the number of relevant clusters of co-expressed genes in terms of disjunct “spots” (assigned with capital letters A–J and IM and MM) and their mutual correlation structure (see Figure 2 and also [9]). Moreover, different areas can be associated with genes specifically overexpressed in one sample class and under expressed in another one (see [6], Figure 2). Instead of plotting profiles of single genes one can indicate the positions of these genes in the map and compare them with its intrinsic structure. We make use of this “mapping” benefit to discuss the behavior of dozens of genes in the five lymphoma and four control classes studied.

For selected objectives, we make use of downstream analysis functions such as gene set enrichment analysis for function mining (see [15]) and weighted topology (wto-) network analysis as described in [11]. Significance of differential expression of single genes and of “spot” clusters of genes was estimated using a shrinkage *t*-test [9,15] implemented in oposSOM [14]. Differential expression and significance maps visualize the degree of differential expression and associated *p*-values in different regions of the map (Supplementary Figure S1).

### 2.3. Pathway Signal Flow (PSF) Analysis

To judge the activity of metabolites not directly transcribed by genes, we make use of the Pathway Signal Flow (PSF) algorithm [16,17,18]. It computes the strength of the signal propagated from pathway input to the output through interactions of pathway component genes, based on their expression values. Here, PSF was used to evaluate the signal flow changes in TCA pathway, for each of the molecular subtypes described above. The reference TCA pathway was taken from the KEGG pathway database (http://www.kegg.jp/kegg/pathway.html, pathway ID: hsa00020). In order to apply the PSF algorithm, we have transformed the cyclic topology of the TCA pathway into a linear one-directional form, by splitting it at the “Oxaloacetate” node, which is presented both as the input and the final output of the pathway (yellow and red nodes of Supplementary Figure S2). The original KEGG TCA pathway is constructed based on KEGG “reaction” interaction type, where each enzyme is connected with direct edges to the substrate and the product. To get one directional signal flow, substrate-enzyme-product reactions were converted to protein-compound-protein interactions (“relations”) with one-directional edges. The PSF algorithm allows to assess the signal flow value both at the output nodes (nodes that do not have any outgoing connections), and at the intermediate nodes (nodes having both incoming and outgoing connections). This gives the opportunity to observe PSF changes in different parts of the TCA cycle by comparing the dynamics of signal flow at intermediate nodes. Note that the PSF is a measure of signaling activity for a certain node of the pathway, which can be either proteins, directly transcribed and translated from their genes (e.g., IDH1) or metabolites, such as 2-oxyglutarate (alias alpha-ketoglutaric acid, α-KG). Hence, the PSF value estimates the activity of pathway products based on their interactions and the expression of the involved genes. It, however, ignores posttranslational factors which in addition can affect the respective products. All PSF data were given in log_10_-scale.

## 3. Results and Discussion

### 3.1. Transcription and DNA Methylation under the Control of Epigenetic Modifiers

#### 3.1.1. Mutual Coupling of Writer/Eraser Activities

**Figure 3:**
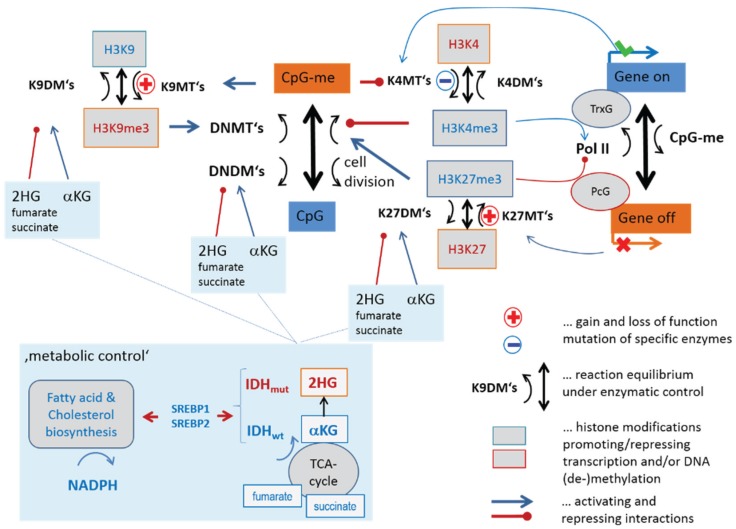
Transcription and DNA (promoter-) methylation under enzymatic control: The scheme summarizes selected regulatory paths affecting histone, CpG-methylation and gene expression via different histone and DNA methylating and demethylating enzymes.

Figure 3 illustrates part of the epigenetic mechanisms regulating gene activity in terms of a simple scheme. They comprise histone modifications, DNA methylation, regulatory interactions and feedback loops between them. We take into account here only three histone modifications, namely trimethylations (me3) of histone subunit H3 at “Lys-4” (K4), “Lys-9” (K9) and “Lys-27” (K27) and CpG methylation of DNA near the promoters of affected genes. Each modification is described as a balance between writing and erasing reactions catalyzed by methyltransferases (for writing methylation marks to histone-lysines and DNA CpG’s) and demethylases (for erasing methylation marks from histone-lysines and DNA-CpG’s), respectively. We will use the abbreviations KDM and KMT for histone lysine demethylases and methyltransferases, respectively, and DNDM and DNMT for DNA-CpG demethylases and methyltransferases, respectively. The scheme provides an idea how histone modifications couple each with another, with DNA methylation and with gene activities.

Gene expression and DNA-methylation is regulated by the battery of enzymes, which either activate or inhibit transcription. For example, trimethylation of H3K4me3 at the promoter is assumed to activate transcription of the respective genes mediated by trithorax group proteins (TrxG). DNA methylation impacts transcription indirectly by reducing the reading capability of TrxG via reduced binding capability of methyltransferases of H3K4me3 [19] and via recruitment of KTMs of H3K9me3 [20]. Complexes containing DNA *de-novo* methyltransferases (DNMT3A/B and L) are assumed to be recruited by H3K9me3 (DNMT3A/B) [21] and repelled by H3K4me3 (DNMT3L) [22]. This mechanism defines a positive feedback loop of *de-novo* DNA methylation via H3K9me3 and DNMT3A/B recruitment and a negative one via H3K4me3 and DNMT3L inhibition. Another positive feedback loop promoting DNA methylation is formed via H3K27me3 and DNMT recruitment [23].

H3K4me3 and H3K27me3 at the promoter act antagonistically, leading to transcriptional activation and repression of the affected genes, respectively. They also have an impact on the regulation of developmental genes in fate decisions [24]. Both processes require reader-writer complexes, namely TrxG and polycomb group proteins (PcG) [25,26], respectively. The latter ones form polycomb repressive complexes (PRC), either PRC1 or PRC2, which act in sequential manner to stably maintain gene repression (see also Figure 1b). PRC2 writes H3K27me3, which is subsequently read by PRC1 creating a silent chromatin state. DNA methylation is also affected by the maintenance methyltransferase DNMT1 to recover methylation marks at the newly synthesized DNA strands after cell division. High methylation and presumably also proliferation rates of the cells require high DNMT1 activities for methylation maintenance [27]. Bivalently (with H3K4me3 and H3K27me3) marked histones give rise to so called poised promoters which are “easy switchable” between active or inactive transcriptional programs by erasing either the H3K27me3 or H3K4me3 marks, respectively.

#### 3.1.2. Mutations of EZH2 and MLL2 Potentially Induce Hypermethylation

**Figure 4:**
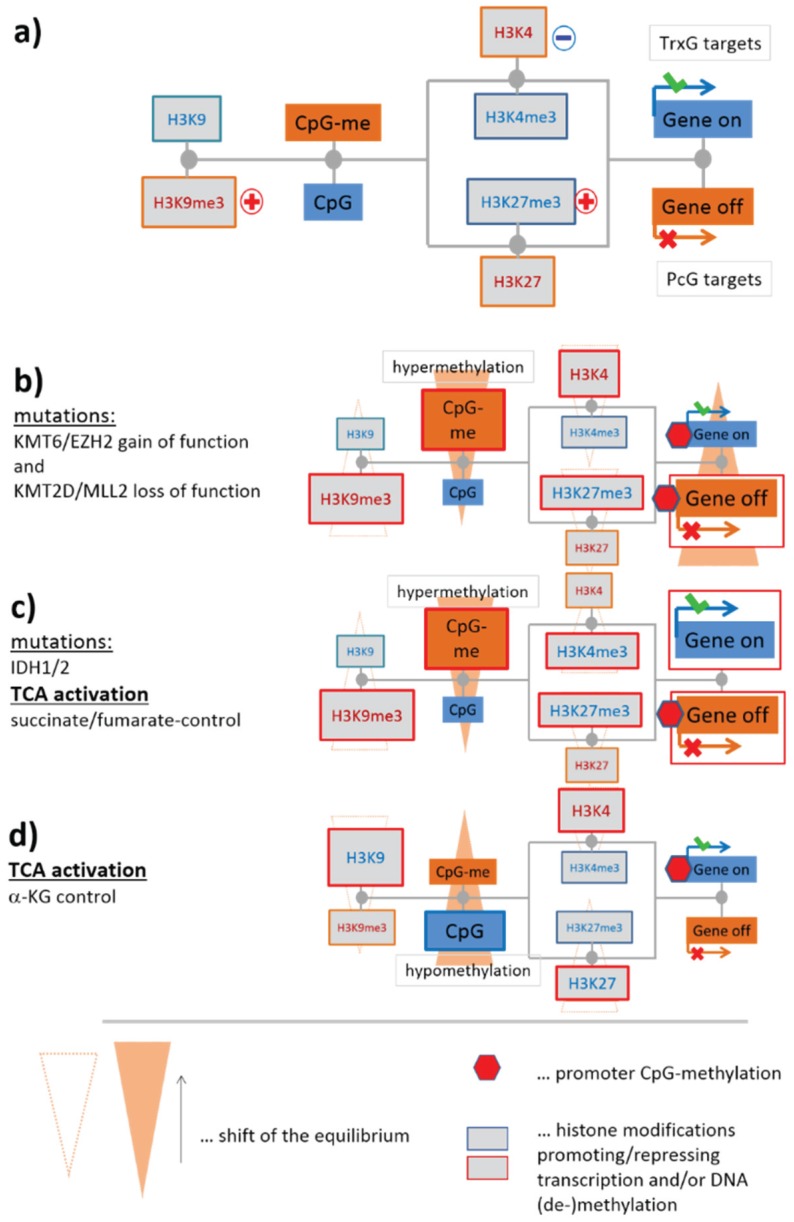
Effect of selected mutations of epigenetic modifiers and of tricarboxylic acid (TCA) metabolism on CpG methylation and gene expression: (**a**) Simplified sketch of the scheme shown in Figure 3. The plusses and the minus indicate gain and loss of function mutations, respectively; (**b**) The scheme suggests that mutations of EZH2 and MLL2 result in DNA-hypermethylation and de-activation of transcription. Alterations are shown by enlarged boxes and red frames in the simplified scheme; (**c**) IDH1 mutations will inhibit a series of KDMs via 2-HG produced by mutated IDH1. It results in hypermethylation and alterations of gene expression in both directions. The same trend is expected for increased TCA-activities with increased levels of fumarate and succinate. Both metabolites inhibit the same modifiers as 2HG; (**d**) TCA activation with increased amounts of α-KG will have the opposite effect as in (**c**): it will demethylate DNA and downregulate expression. Note that global changes of methylation (as indicated by “CpG-me”) are paralleled by local ones due to the hypothesized alterations of gene expressions: red marks indicate promoter methylations in (**b**–**d**) recruited by polycomb group (PcG)-repressed and/or trithorax group (TrxG)-deactivated genes.

Genome screening in patients with lymphoma have detected a series of mutations in genes involved in the epigenetic regulation of transcription [28,29,30,31,32,33] (see Table 1 below). Mutations of critical role in lymphomagenesis occur in genes transcribing epigenetic modifiers, such as KMT6 (alias EZH2, being a K27MT) leading to a gain of function preferentially in DLBCL [28,34,35], and KMT2B/2D (alias MLL2, being a K4MT) giving rise to its loss of function in follicular lymphoma (FL), as well as in DLBCL [36] (see red plus and blue minus signs in Figure 3 and also in the simplified scheme shown in Figure 4a). Our scheme suggests, in this particular case, an increase in DNA methylation and a decrease in gene activity based on the altered activities of these enzymes (see Figure 4b for illustration). Resulting hypermethylation will affect PcG- and TrxG-related genes as well. Indeed, net-hypermethylation of PRC2 target genes (polycomb repressive complex 2) was reported for DLBCL, compared with healthy B and GCB cells [6]. This methylation change is induced by hyper-trimethylation of H3K27 as found recently in enzyme activity experiments [37].

#### 3.1.3. Epigenetics under Metabolic Control

IDH1 and IDH2 (for short IDH1/2) catalyze the interconversion of isocitrate and α-ketoglutarate (α-KG alias 2-oxoglutarate). α-KG is a tricarboxylic acid (TCA) cycle intermediate and an essential cofactor for many enzymes, including Jumonji-domain containing (JmjC) KDMs such as KDM2A, 4B/C, 5C and TET-family DNDMs [38]. Cancer-associated IDH1/2 mutations alter the enzymes such that they reduce α-KG to the structurally similar metabolite (R)-2-hydroxyglutarate (2HG). α-KG generates nicotinamide adenine dinucleotide phosphate (NADPH), whereas mutant IDH1/2 converts α-KG into 2HG and consumes the reducing agent NADPH. 2HG has been shown to inhibit JmjC-KDMs and TET-DNDMs leading to aberrant epigenetic modifications in tumor cells [39,40,41]. The inhibitory effect of 2HG is expected to have a similar effect on our regulatory network, as do the mutations of EZH2 and MLL2 (Figure 4c). This similarity presumably explains the observation that both cancer entities share a high fraction of PRC2-related and hypermethylated genes [6]. Mutations of IDH1/2 are frequent events in tumors such as gliomas [3,42,43] and leukemia [44]. IDH1/2 mutations are, however, rather scarce in lymphomas [45] and cannot account for such parallel effects.

One can, however, hypothesize that intermediate products of the TCA-cycle, such as succinate and fumarate, have a similar effect on epigenetics like 2-HG by thus proving a possibly explanation of the observed effects [38] (Figure 4c). Recall that widespread metabolic alterations enable tumor cells to continuously survive and proliferate in certain tumor microenvironments [46]. Among lymphomas, especially BL but partly also IntL are characterized by high proliferative activity, strongly activated energy metabolism and mitochondrial function, which were often paralleled by activated c-MYC expression [10,47]. Such massive metabolic changes suggest interference with epigenetic regulation via modifying enzymes responding to metabolites. Activating interaction, e.g., due to high abundance of α-KG is expected to demethylate histone lysines and DNA CpGs and to activate expression, *i.e*., alterations not corresponding to the observed ones (Figure 4d). However, other intermediate products of the TCA-cycle, namely succinate and fumarate, are shown to counteract α-KG. Their enhanced production in metabolically activated lymphoma subtypes possibly explains the observed trend [38] (Figure 4c).

**Table 1:** Chromatin modifiers with possible relevance for lymphoma: an overview.

Target	Type^1^	Enzyme	Alias	Mark	txn^2^	mut^3^	DEx^4^	Spot^5^	Comment^6^
	Writer Eraser Reader			Me	act. repr.		LvsBc^4^		
**H3K4**	W	**KMT2A**	MLL		act		−	IJ	TrxG:MLL complex
		**KMT2B**	MLL2, KMT2D		act	x	−	J	loss of function in DLBCL/FL, TrxG:MLL complex
		**KMT2F**	SETD1A		act		−	(H)	
		**KMT2G**	SETD1B	Me3	act		−	H	
		KMT3C *	SMYD2	Me2/Me3	act		x	(MM)	
		**KMT3E**	SMYD3	Me2/Me3	act		+	(D)	
		**PRDM9**	MSBP3. PFM6	Me3	act		−	(I)	
		SETMAR *	METNASE		act		x	B	
	E	KDM1A *	LSD1, AOF2	Me1/Me2	rep		+	B	“gene body cleaner”
		**KDM5A**	JARID1A, RBBP2	Me2/Me3	rep		x	(A)	JmjC, “gene body cleaner”
		**KDM5B**	JARID1B, PLU1		rep		−	(F)	JmjC
		**KDM5C**	JARID1C, SMCX		rep		+	(I)	JmjC
**H3K9**	W	KMT1C *	EHMT2, G9A	Me1/Me2	rep		x	(B)	
		KMT1D *	EHMT1, GLP	Me1/Me2	rep		+	(D)	
		**KMT1E**	SETDB1	Me3	rep		x	(I)	
		KMT6(A) *	EZH2		rep	+	+	D	gain of function in cancer/DLBCL/FL, PRC2 complex
		**KMT8**	PRDM2, RIZ		rep		−	(D)	Missense mutation in DLBCL
	E	KDM1A *	LSD1, AOF2		act		+	B	
		**KDM3A**	JMJD1, TSGA	Me1/Me2	act		−	(IM)	JmjC
		**KDM3B**	JHDM2B		act		x	(C)	JmjC
		KDM4A *	JMJD2	Me3	act		−	(J)	JmjC
		**KDM4B**	JHDM3B	Me3	act		−	J	JmjC
		KDM4C *	JHDM3C	Me3	act		−	I	JmjC
		**KDM4D**	JMJD2D	Me2/Me3	act		x	(B)	JmjC
		KDM7A *	JHDM1D	Me2	act		−	H	JmjC
		**MINA**	MDIG, ROX	Me3	act		+	(B)	
**H3K27**	W	KMT1C *	EHMT2, G9A		rep		x	(B)	
		KMT1D *	EHMT1, GLP		rep		+	(D)	
		KMT6(A) *	EZH2		rep	+	+	D	gain of function in cancer/DLBCL/FL, PRC2 complex
		**KMT6B**	EZH1		rep		−	J	PRC2 complex
		**WHSC1**	NSD2, MMSET		rep		+	D	mutated in BL and MCL, opens chromatin
	E	**KDM6A**	UTX	Me2/Me3	act		−	(IM)	
		**KDM6B**	JMJD3	Me2/Me3	act		−	J	involved in inflamma-tory response, JmjC
		KDM7A *	JHDM1D	Me2	act	x	−	H	JmjC
**H3K36**	E	**KDM2A**	FBXL11, JHDM1A	Me2			−	(H)	JmjC
		KDM4A *	JMJD2	Me3	rep		−	(J)	JmjC
		KDM4C *	JHDM3C	Me3	rep		−	I	JmjC
		**KDM8**	JMJD5	Me2	rep		x	(B)	JmjC
	W	**KMT2H**	ASH1L		act		−	(I)	
		**KMT3A**	SETD2, SET2	Me3	act		−	J	recruits MMR
		**KMT3B**	NSD1, STO				−	(J)	
		KMT3C *	SMYD2	Me2	act		x	(MM)	
		SETMAR *	METNASE	Me2	act		x	B	
**H3K79**	W	**KMT4**	DOT1L		act	x	x	(MM)	Loss of function in Lymphoma
**DNA**	W	**DNMT1**			rep		+	D	maintenance
		**DNMT3A**			rep		x	(D)	*de novo* methylation
		**DNMT3B**			rep		+	B	*de novo* methylation
		**DNMT3L**			rep		+	(D)	Induces *de novo* DNA methylation by recruitment or activation of DNMT3
	E	**TET3**			act		−	I	
	R, E	**MBD2**			act/rep		−	(I)	mediates CpG-methylation signal

^1^: Epigenetic writers add the covalent modification to either histone tails or the DNA. Here we consider only histone lysine methyltransferases (KMTs) and DNA methyltransferases (DNMTs). Epigenetic erasers catalyze the removal of epigenetic marks, e.g., to alter gene expression. Here we consider only histone lysine (KDMs) and DNA (DNDMs) demethylases. Epigenetic readers possess effector domains and recognize and bind to modified residues. Many “classical” transcription factors (that “read” special DNA binding motifs) are also epigenetic readers because their binding to DNA is also governed by epigenetic marks (see also [1,48] and Figure 1b); ^2^: expected net effect on the transcriptional activity of the affected genes. In general there is no one-to-one relation between a certain epigenetic modifier and the change of gene expression. Combinations of modifiers and their marks give rise to a large variety of options (called also chromatin code). Here we assign the proposed effects of chromatin marks on gene expression according to GeneCards (www.genecards.org); ^3^: activating/gain of function (+) or deactivating/loss of function (x) mutation observed in lymphoma; ^4^: differential expression with respect to B cells: +…up; − …down; x…indifferent; ^5^: spot cluster: e.g., A… gene belongs to spot A; (A)…gene is found near spot A in the map; spot characteristics: B,C,D: up in BL and down in DLBCL/FL; F: up in FL; I: up in BCL and MM and down in BL and partly DLBCL; J: up in B and GCB cells and down in lymphoma; H: up in B cells, tonsils and FL, down in BL; ^6^: mutation data and assignments to lymphoma classes were taken from [28,29,30,31,32]; *: enzymes marked with asterisks perform multiple roles by catalyzing more than one lysine side chain.

In summary, our simple scheme predicts the activation of repressed PcG-related genes paralleled by DNA-hypermethylation after mutations of the genes EZH2 and/or MLL2, which both code lysine-methyltransferases. Mutations of IDH1/2 or an increased TCA-activity are suggested to have a similar effect on histone and DNA methylation. However IDH1/2 mutations are scarce in lymphoma suggesting alternative mechanisms that couple metabolism with epigenetics.

### 3.2. Expression Cartography of Epigenetic Modifiers

In this subsection, we aim to verify the predictions made above. Using lymphoma expression data we will systematically monitor the expression levels of about fifty methylating and demethylating enzymes in different lymphoma subtypes and healthy controls to document their heterogeneity in regulating gene activity (see Table 1 for an overview). Note that the enzymatic activity is modulated by a series of post-transcriptional and -translational factors (such as posttranslational modifications, local accessibilities and concentrations of cofactors), which are beyond our data.

#### 3.2.1. SOM Expression Map of Epigenetic Modifiers

For a holistic view, we make use of SOM-portayal method. It locates the genes coding the modifying enzymes into a quadratic map, which visualizes the “expression landscape” of the lymphoma-cohort studied. In this map, co-expressed genes with similar expression profiles cluster together within spot-like regions (assigned by capital letters A–J and MM, IM; see Figure 2) thus allowing to deduce the expression characteristics of a gene from its location in the map [10,11].

**Figure 5:**
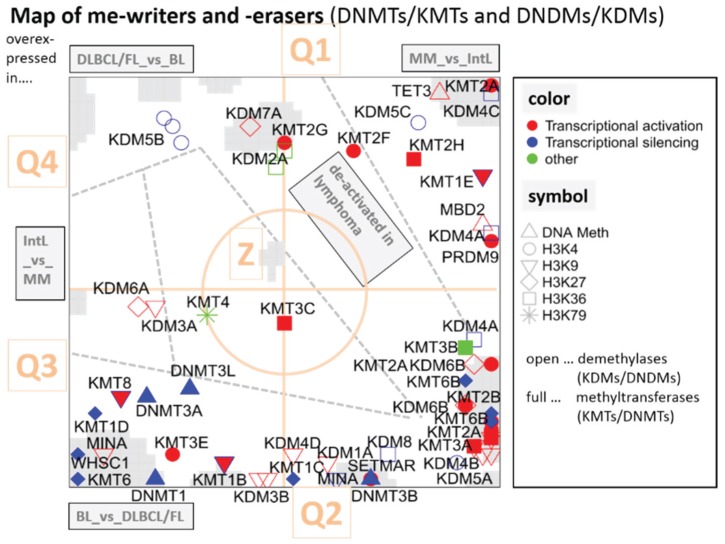
Mapping of writers and erasers of epigenetic methylation marks into the gene expression landscape (DexSOM) of lymphoma. The map is segmented into regions of specific differential expression between the lymphoma classes by dashed lines. The mode of differential expression is indicated in the grey boxes (e.g., “IntL_*vs.*_MM” contains genes upregulated in IntL compared with MM). So-called spot-clusters of differentially expressed genes are grey-colored. The map is further divided into four quadrants Q1–Q4 and a central region “Z” (see Figure 2 for segmentation of the SOM). Epigenetic modifiers are labeled as shown in the right part of the figure. A few genes are redundant because more than one probe set interrogate them (e.g., KDM4A, KDM6B, KMT6B). The redundant probe sets are located in close proximity reflecting strongly correlated expression profiles (see Table 1 for assignment of the enzymes).

**Figure 6:**
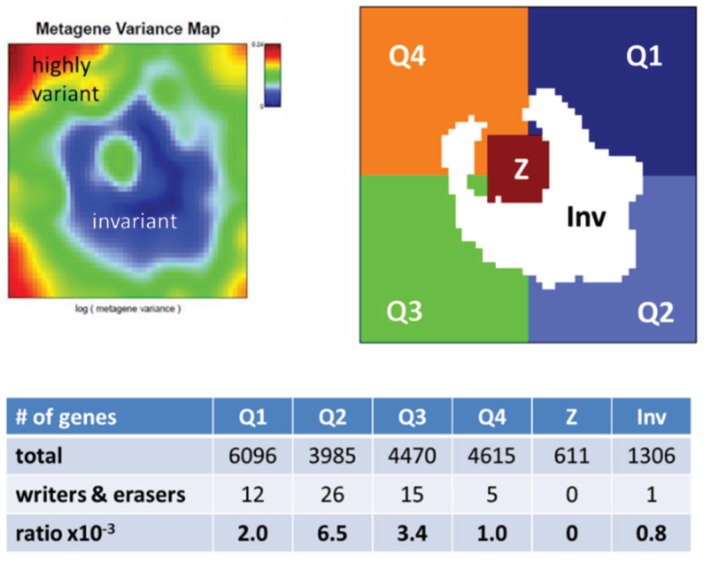
Enrichment characteristics of writers and erasers of histone and DNA methylation marks in the expression SOM. We segmented the map as shown in Figure 2 into four quadrants Q1–Q4 and a central area Z. In addition, we separately considered genes with invariant expression profiles, which populate the blue area in the variance map (Inv). Then we counted the number of genes and of writers and erasers of methylation marks in each of the areas. These modifiers were strongly enriched in Q2, on intermediate levels in Q1 and Q3 and on low levels in Q4, Inv and Z.

The overview map shown in Figure 5 summarizes the location of genes encoding writers and erasers of histone-lysine and of DNA-CpG methylation marks listed in Table 1. For sake of simplicity we segmented the map into four quadrants Q1–Q4 containing genes differentially up- and down-regulated in selected classes and a central region Z containing mostly invariant genes (Figure 2). Interestingly, the genes encoding epigenetic modifiers strongly accumulate in Q2, to a less degree in Q1 and Q3, but they are almost absent in Q4 (Figure 6). This asymmetric distribution reflects the fact that gene expression of the majority of methylating and demethylating enzymes is either up- or down-regulated in lymphoma compared with B cells and/or activated in BL compared with DLBCL and FL. Enzymes up-regulated in DLBCL and/or FL compared with BL are however rather scarce.

According to the histone code hypothesis numerous of histone modifications are assumed to regulate gene expression of the associated genes. In Figure 5, we color coded the assumed effect on transcription by symbols being red for activation, blue for silencing and green for unknown effect. Enzymes promoting gene expression are slightly enriched in Q1, which contains genes down-regulated in lymphoma. Thus, activating marks in Q1 correspond to the expression in B cells. On the other hand, Q2 and Q3 are more puzzling, as those reflect no preference for activating and de-activating marks.

#### 3.2.2. DNA Methylating Enzymes: DNA-MTs and -DMs

**Figure 7:**
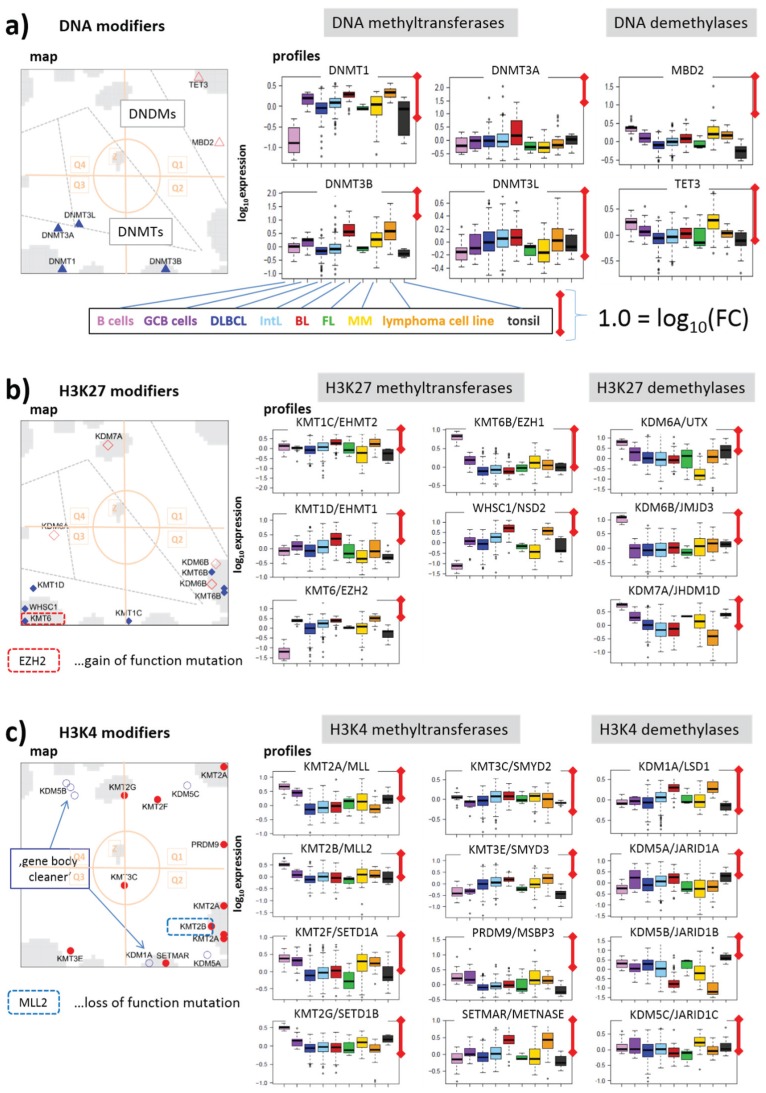

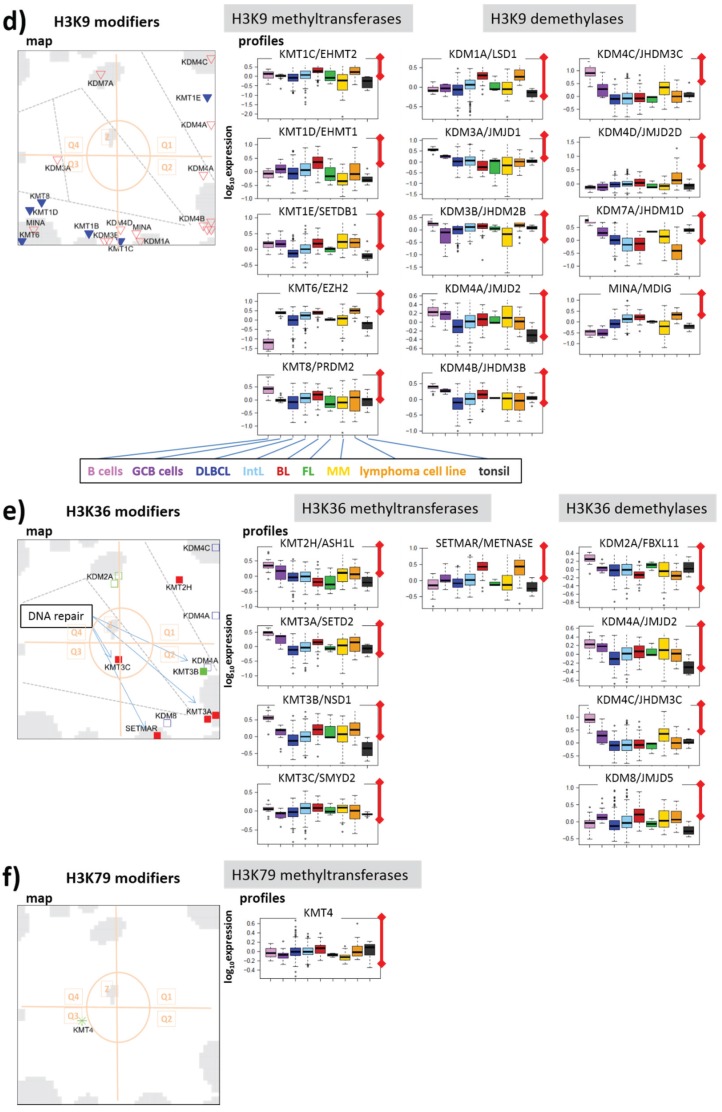
Group-wise mapping of writers and erasers of methylation marks at CpGs and lysine side chains of histone subunit H3 and their expression profiles: (**a**) DNA, (**b**) H3K4, (**c**) H3K27, (**d**) H3K9, (**e**) H3K36 and (**f**) H3K79. The symbols and their coloring are assigned in Figure 5. Modifiers (de-)marking more than one lysine-type are shown several times. Note the different scales of ordinate-axes. The length of the red scale-bar refers to a fold change (FC) between two expression values of one order of magnitude.

DNA methyltransferases and demethylases accumulate in opposite corners of the map shown in Figure 7a (Q3 *versus* Q1) being up- and down-regulated in lymphoma compared with B cells. The *de-novo* methyltransferases DNMT3A,B,L show only moderate effect also between GCB and B cells in agreement with previous results [49]. All of them, but especially DNMT3A, have a high gene activity in BL and a relatively low one in the other lymphoma subtypes. The maintenance methyltransferase DNMT1, on the other hand is strongly up-regulated in lymphoma and GCB cells showing also maximum activity in BL. Previous results suggested that DNMT1 carries out multiple functions in support of the GC phenotype including chromatin condensation/de-condensation, maintenance of genomic DNA methylation and also double strand DNA break repair [49].

Note that proliferative activity is extraordinarily high in BL, also requiring high activities of CpG methylation maintenance and DNA repair processes. Demethylases, on the other hand, are on highest expression levels in MM revealing a strong antagonism of DNA methylation and demethylation between MM and DLBCL (and BL), between BL and DLBCL and partly between B and GCB cells. Note that DNA methylation is not a simple sum of DNMT activities. A comparison of the epigenomes between normal and cancerous stem cells, and between pluripotent and differentiated states shows that the presence of at least two DNMTs is required for differential DNA methylation effects [50]. Moreover DNA (de-) methylating enzymes operate often in concert with histone modifications.

DNA demethylases of the TET-family play an important role in fine regulation of DNA methylation. Their inactivation leads to the establishment of DNA hypermethylation phenotype [51]. TET3 locates near spot I meaning low expression levels in DLBCL and IntL but relatively strong ones in MM and B cells. This inactivation in DLBCL and IntL indeed accompanies with aberrant methylation patterns partly resembling methylator phenotypes observed in other cancer types such as colorectal cancer and glioma [6]. The possible role of TET-family proteins in coupling mechanisms with the TCA-metabolism will be discussed below. MBD2 is a methyl-CpG-reader that has been reported to be both a transcriptional repressor and a DNDM [52]. In lymphoma it shows a similar expression profile as TET3.

#### 3.2.3. Histone K27MTs and DMs

Figure 7b–f disentangles the KMTs and KDMs according to the position of the methylated/de-methylated lysines in the H3 subunit. Firstly, we see strong activation of EZH2/KMT6(A) in all lymphoma subtypes compared with B cells (Figure 7b). A high fraction of DLBCL and of grade 3 FL harbors EZH2 mutations, suggesting that these mutations are likely to be early lesions during lymphomagenesis. EZH2 gain of function mutation presumably promotes malignant transformation by repressing both anti-proliferative and differentiation-inducing programs [35,53,54,55]. In general, EZH2 represses proliferation checkpoint genes. In GCB cells EZH2 bivalent chromatin domains are built at key regulatory regions to temporarily repress GCB cell differentiation. These physiological effects are amplified by somatic mutations through enhanced silencing of EZH2 targets leading to malignant transformation into highly proliferative lymphoma types [55]. Our data show that EZH2 expression in all lymphoma subtypes except for BL is decreased compared with GCB cells, but it is increased compared with B cells.

EZH1/KMT6B, another H3K27-methyltransferase (and a homolog of EZH2) and the KDMs UTX/KDM6A and JMJD3/KDM6B show roughly antagonistic profiles compared with EZH2 as they are strongly deactivated in lymphoma. KDM6A and B play an important role in the differentiation of tissues from embryonic stem cells (ESC), where their deactivation impairs differentiation [56,57]. Interestingly, JMJD3 activates bivalent genes in response to lipopolysaccharide inflammatory stimulus in macrophages [57,58,59]. Moreover, somatic mutations of KDM6A have been found in a number of cancer types indicating the importance of this enzyme in tumorigenesis. The antagonistic changes of EZH2 and UTX in lymphoma suggest that the methyltransferase and demethylase act in a concerted fashion and shift the methylation equilibrium towards trimethylated H3K27me3, which promotes repressive transcriptional states.

EZH1 safeguards ESC identities and maintains repression in resting cells [60]. It is more abundant in non-proliferative adult organs and acts transcriptionally as antagonist of EZH2, which *de-novo* establishes H3K27me3 in dividing cells [61,62,63]. Our data thus support the view that activation of EZH2 in lymphoma “over-represses” suppressors of proliferative programs, whereas de-activation of EZH1 “under-represses” maintenance suppressor of resting cells presumably thus destabilizing their state.

WHSC1, another K27MT, changes in lymphoma in a similar way as EZH2. It is frequently mutated in mantle cell lymphoma [64] and partly also in BL [65]. It has been suggested that WHSC1 mutations in these lymphoma types were associated with open chromatin states in their cell(s) of origin [65]. Note that WHSC1 and other genes located in Q3 (spot D in the DexSOM) are up-regulated in BL and partly GCB cells thus supporting this view (see also [6], where we assign these genes to euchromatin states in BL). WHSC1 is also activated in MM where it is thought to open chromatin structure [66]. Solely the enzyme KDM7A is located in Q1 (spot H) due to the fact that it is specifically activated in FL and MM. This enzyme de-methylates H3K27me2.

In summary, the expression profiles of H3K27 (de)-methylating enzymes reflect concerted deregulation of repressed (including also poised) genes in lymphoma. These genes seem to become “over-repressed”, which presumably results in a loss of plasticity of cellular programs. In consequence, the cells become unable to return into an active state as required for healthy GCB cell function.

#### 3.2.4. Histone K4MTs and DMs

Genes encoding methyltransferases for Lys-4 accumulate in Q2 (mainly in spot J) thus resembling the profiles of a series of K27MTs and especially K27DMs (see Figure 7b,c for comparison). This region also contains KMT2B (MLL2) frequently carrying a loss of function mutation in about 30% of DLBCL and 90% of FL patients [36,67]. Down-regulation of this gene is indeed observed in lymphoma (Figure 7c), which is assumed to act as a central tumor suppressor [36] and to de-activate TrxG-related genes. Another gene of the MLL-group, KMT2A (MLL) shows a virtually identical profile suggesting similar function. KMT2A is targeted by chromosomal translocations deactivating this gene on Chromosome 11q23 in lymphoma [68].

K4 (de-) methyltransferases are depleted in Q3 and partly enriched in Q1 in sharp contrast to K27 (de-) methyltransferases reflecting the partly antagonistic role of both types of modifiers in either activating or repressing transcription. Only KMT3E is found in Q3 (near spot D). KMT3E (SMYD3) knockdown causes cell cycle arrest and induction of apoptosis [69]. Hence, its up-regulation in lymphoma and especially in BL associates with the opposite effects leading to increased proliferation and anti-apoptotic “cancer hallmark” activities.

Interestingly, the H3K4 demethylase KDM5B (alias JARID1B) is among the very few genes found in Q4, which contains genes specifically up-regulated in DLBCL and FL. KDM5B acts as “gene body cleaner” by focusing H3K4me3 methylation near promoters and enhancers of bivalent (*i.e*., weakly transcribed) genes by demethylating their gene bodies during embryonic stem cell self-renewal and differentiation [70]. This mechanism ensures correct expression of the affected genes. The H3K4 demethylase KDM1A (alias LSD1) also demethylates H3K4me3 in gene bodies, however, in inactive genes. Interestingly, KDM1A is located in Q2 (spot B), which roughly antagonistically switches with respect to Q4 (spot F) co-expressed with KDM5B. These results suggest that stemness genes with bivalently marked promoters co-regulate with KDM5B activity because their functionality is maintained by this enzyme, while genes repressed in ESC differentiation co-regulate with KDM1A activity. Proper function of these enzymes maintains developmental genes in their bivalent-active or repressive state. Another study showed that ectopic expression of KDM5B, promotes the epithelial-mesenchymal transition of cancer cells [71].

In summary, writers and erasers of H3K4me3 tend to show an antagonistic behavior compared with the respective H3K27-modifiers, which corresponds to their mostly antagonistic effect on gene expression. Up-regulation of K4DMs and deactivation of K4MTs seems to lead to under-activation of tumor suppressors controlling, e.g., apoptosis and proliferation.

#### 3.2.5. Histone K9MTs and DMs

H3K9me3 promotes CpG-methylation and gene deactivation (Figure 3). The distribution of H3K9 (de-) methylating enzymes in the DexSOM shares similarities with that of H3K27me3 representing the second deactivating mark considered here (compare Figure 7b,d). Particularly, K9MTs accumulate in Q3 (spot D) up-regulated in lymphoma and especially in BL. Note that part of the modifiers (e.g., EZH2 /KMT6) affect both H3K9 and H3K27. Interestingly, K9DMs tend to occupy a wide area in the map ranging from Q3 (spots C) over Q2 (especially spots B and J) to Q1 (spot I) thus showing activation (Q3) and deactivation (Q1 and Q2) in lymphoma compared with B cells (see also the profiles in Figure 7d). For example, KDM4D (Q3) demethylating H3K9me3 [72] specifically up-regulates in BL. Other members of the KDM4-family—KDM4B (spot J), KDM4A and KDM4C (spot I)—are deactivated in lymphoma thus presumably promoting H3K9me3 and DNA CpG methylation. Contrarily, these enzymes are overexpressed in other cancers such as breast, colorectal, lung and prostate cancer because they are required for efficient cancer cell growth [73,74]. KDM4C shows the most pronounced effect among them, being active in B cells and MM, on intermediate level in GCB cells and on lowest level in DLBCL, thus suggesting a certain role in DNA-hypermethylation observed in lymphoma. The activity of this enzyme clearly anticorrelates with the energy metabolism possibly due to transcription factor (TF) regulation via SREBP1 and/or intermediates of the TCA cycle inhibiting its activity (see below).

#### 3.2.6. Histone K36MTs and DMs

Part of the KDM4-family also demethylates H3K36me3 thus activating expression according to the histone code because of its role in transcriptional elongation. This dual function is thought to repress aberrant transcription [75]. H3K36 marks distribute over the gene body and perform fine tuning of expression by interacting with RNA Polymerase II. They also play a role in nucleosome positioning, alternative splicing and exon activation [76]. Interestingly, H3K36me3 is required as reading mark for DNA repair proteins by acting as chromatin switch, which makes DNA accessible for double strand repair [77]. For example, high levels of KMT3A (SETD2) support errorless homologous DNA repair in human cells [78]. KMT3A expression down-regulates in lymphoma and especially in DLBCL (Figure 7c, Q2, spot J) suggesting reduced potential for DNA repair. On the other hand, the associated demethylase KDM4A counteracting KMT3A shows a similar expression profile indicating a more complex effect. Other members of the KMT3 family (KMT3B and KMT3C) co-regulate with KMT3A thus promoting H3K36me3 demethylation in lymphoma. In summary, K36MTs and K36DMs both accumulate in Q1 and Q2 with homogeneous expression profiles reflecting their down regulation in lymphoma compared with B cells.

#### 3.2.7. Histone K79MT

KMT4-mediated H3K79 di- and tri-methylation is essential for embryogenesis and hematopoiesis. The sole enzyme responsible for H3K79 methylation considered here is KMT4 (DOT1L) promoting transcription by stimulating its elongation phase [79]. KMT4 is located in the central zone of the map (Z) near spot MM (Figure 7f) up-regulated only in MM suggesting a specific role of this enzyme in this lymphoma class. DOT1L has also been implicated in the development of mixed lineage leukemia (MLL)-rearranged leukemia, where mistargeting of DOT1L causes aberrant H3K79 methylation.

### 3.3. Expression Cartography of Chromatin Remodeling Complexes

**Figure 8:**
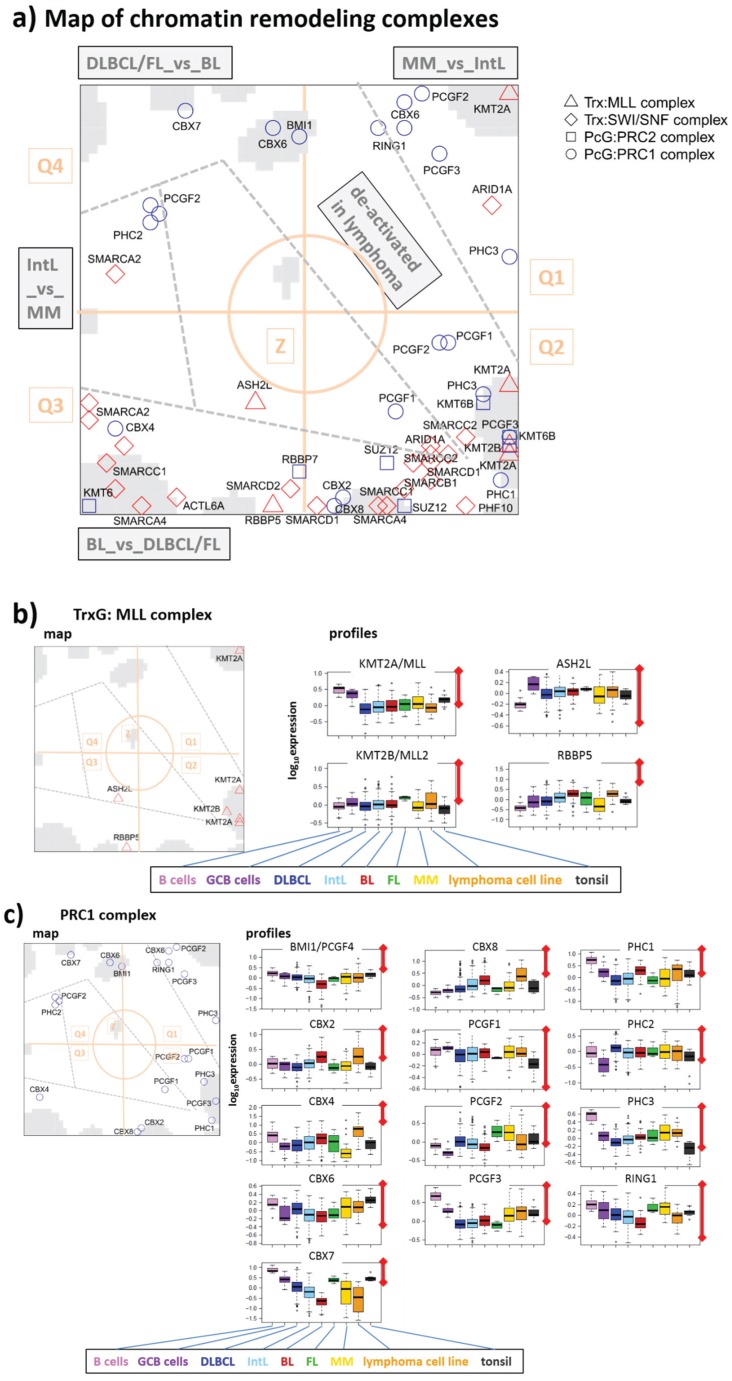

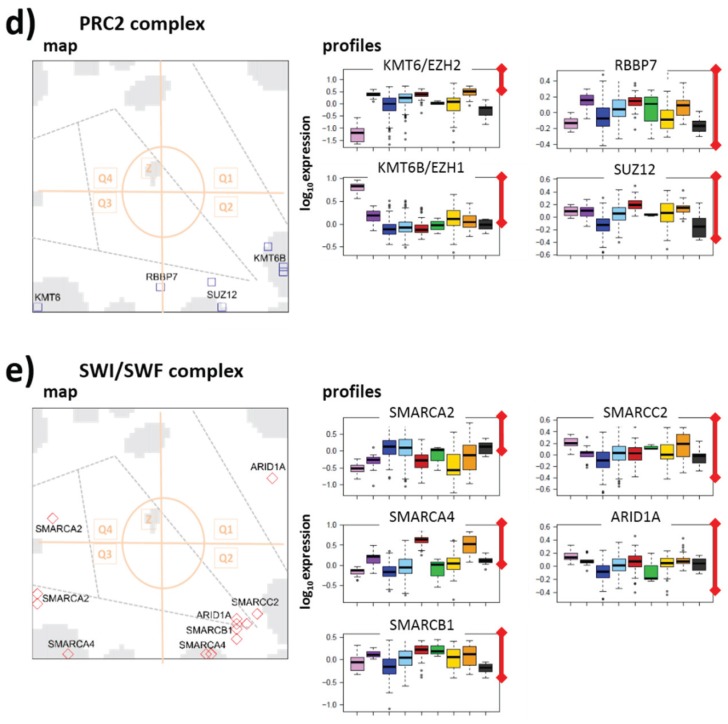
Map of ingredient-genes of chromatin modifying complexes: (**a**) overview map, (**b**) TrxG/MLL-, (**c**) PRC1-, (**d**) PRC2-, and (**e**) ATP-dependent chromatin remodeling SWI/SWF complexes, respectively.

A large fraction of modifiers discussed in the previous subsection act in concert each with another and form different kinds of functional complexes together with writers. In this subsection we regrouped the enzymes and complemented them with relevant writer-proteins in a complex-related order to discover their expression profiles in the cohort studied. The overview map shown in Figure 8a reveals accumulation of the complex-related genes (except for PRC1- related genes) in Q2 and Q3 reflecting a further narrowing of the regulatory space compared with the full set of modifying enzymes (compare with Figure 5a). Recall that Q2 (especially spot J) collects enzymes down-regulated in lymphoma, whereas Q3 contains genes up-regulated in lymphoma with high expression levels in BL and relatively low levels in DLBCL and FL. Hence, the map reflects a dual antagonism of activation/de-activation patterns, namely (i) up in lymphoma and down in B cells and *vice versa* and (ii) up in BL and down in DLBCL and FL where, however, the antagonistic mode is almost lacking (see also the correlation maps in Figure 2c).

#### 3.3.1. TrxG/MLL Complex

TrxG/MLL is a reader-writer complex to complete H3K4me3 methylation and to activate expression [63,80]. The retinoblastoma binding protein 5 (RBBP5) and the Ash2L protein are conserved subunits of the MLL complex, which form a heterodimer with intrinsic methyltransferase activity required for methylation of H3K4 [81,82]. Expression of both compounds is high in proliferative GCB cells and BL, and low in B cells, MM and partly DLBCL (Figure 8b). Their profiles partly diverge from that of the KMTs showing partly antagonistic changes.

#### 3.3.2. PRC1 Complex

The PRC1 complex stabilizes gene repression established by PRC2 beforehand (see below). Main compounds are chromobox homolog (CBX)-family proteins (alias heterochromatin protein, HP1), which are essential for heterochromatin formation and stabilization. Part of them (CBX2, 4 and 8) are located in Q3 and partly Q2, whereas the others (CBX6 and 7) are found in the opposite half of the map (Q4 and Q1) revealing either up-regulation in BL and down-regulation in DLBCL and FL or *vice versa*, respectively (Figure 8c). Large amounts of CBX proteins seem to serve as reservoir for heterochromatin formation to bind to the nucleosomes upon request and stabilize repressive chromatin states during cell differentiation [83]. The antagonism of CBX expression between BL and DLBCL suggest chromatin remodeling between euchromatin in BL and heterochromatin in DLBCL as hypothesized in [6]. CBX2 binds to genes deactivated by H3K27me3 [84]. Such genes accumulate in spot B together with CBX2 indicating coregulation and particularly deactivation in DLBCL (see next subsection). Elevated expression of CBX7 and of BMI1 (alias PCGF4) in DLBCL and FL (Q4) are related to aberrant regulatory programs inducing high-grade tumor transformation and chemotherapy resistance [53]. Other compounds of PRC1 are polycomb group RING finger (PCGF) and polyhomeotic homolog (PHC) proteins accumulating in Q1 and Q2 thus indicating down-regulation in GCB-derived lymphoma. Importantly, different ingredients of PRC1 fulfill different roles in chromatin condensation and they bind also to different genomic loci thus defining different subgroups of PRC1 [84], which possibly explains the different profiles of CBX and of PCGF/PHC proteins.

#### 3.3.3. PRC2 Complex

PRC2 catalyzes methylation of H3K27me3 through its “enhancer of zeste” (EZH) constituents (see above). Other compounds are SUZ12 and RBBP7 both required for the establishment of specific expression programs needed for differentiation of embryonic stem cells [85]. All PRC2 compounds studied were found in Q2 and Q3 (Figure 8d), which well correspond to the distribution of CBX-proteins discussed above: PRC2 genes in Q2 and Q3 are *de-novo* and temporarily repressed in DLBCL by H3K27me3. Afterwards they then transform into permanently repressed heterochromatin by CBX-PRC1 binding. Note also that SUZ12 and PRC2 targets strongly enrich in Q1 and Q4 [6], containing genes, which antagonistically switch compared with Q2 and Q3 genes (see Figure 2c). This suggests that PRC2 and SUZ12 targets up-regulate in DLBCL owing to “over-supressing” their regulators.

Note that during normal B-lymphocyte differentiation, expression of PRC1 and PRC2 genes show a restricted, stage-specific pattern: PRC1 genes are primarily detected among resting B cells in the GC mantle zone and in non-dividing centrocytes of the GC. Contrarily, PRC1 genes become silenced in proliferating follicular centroblasts, which then express the PRC2 genes instead. Lymphomas generally lose such a mutually exclusive expression pattern. Altered expression of PRC1 and PRC2 genes is a general theme in lymphomas suggesting essential regulatory roles of PRC1 and PRC2 in both normal B-lymphocyte development and lymphoma pathogenesis [53].

#### 3.3.4. SWI/SWF Complex

The SWI/SWF complex belongs to the ATP-dependent chromatin remodeling complexes. They remodel chromatin (and particularly the packing of the nucleosomes) to make it DNA accessible during transcription, replication and DNA repair [86]. Particularly, the SWI/SWF-complex accomplishes tasks such as nucleosome unwrapping, mobilization, ejection and histone dimer exchange [86]. Notably, the ingredient genes of this complex accumulate also in Q2 and Q3 (Figure 8e) together with PRC2 and CBX-PRC1 genes. Hence, SWI/SWF genes regulate in concert with the main regulatory modes differentiating BL and DLBCL and, partly also lymphoma and healthy B cells. Most of the SWI/SWF compounds are highly expressed in BL and weakly expressed in DLBCL, which also supports our view of extended remodeling from open euchromatin to closed heterochromatin between both lymphoma subtypes [6]. Possibly euchromatin is maintained in BL by high activity of SWI/SWF-compounds and low activity of CBX-PRC1-compounds (see above). One of the genes coding SWI/SWF-compounds, SMARCA4, is frequently mutated in BL [28,29]. It is strongly overexpressed in this subtype (Figure 8e). Targets inhibited by SMACA4 [87] are found in Q4 to be down-regulated in BL [6].

### 3.4. Energy Metabolism Couples with Epigenetics in Lymphoma?

Mutations and resulting aberrant expression of metabolic enzymes such as IDH1/2, fumarate hydratase (FH) and succinate dehydrogenase (SDH) are possible drivers of tumorigenesis [38]. α-KG, a metabolic intermediate of the TCA cycle plays critical roles not only for energy metabolism but also as a precursor of glutamine formation, for amino acid synthesis, as a nitrogen transporter for the urea cycle and ammonia detoxification, and as a co-substrate for α-KG-dependent dioxygenases (see [38] for a detailed discussion). Among these enzymes one finds JmjC KDMs and the TET-family DNDMs. Their deregulation contributes to tumorigenesis via epigenetic effects.

**Figure 9:**
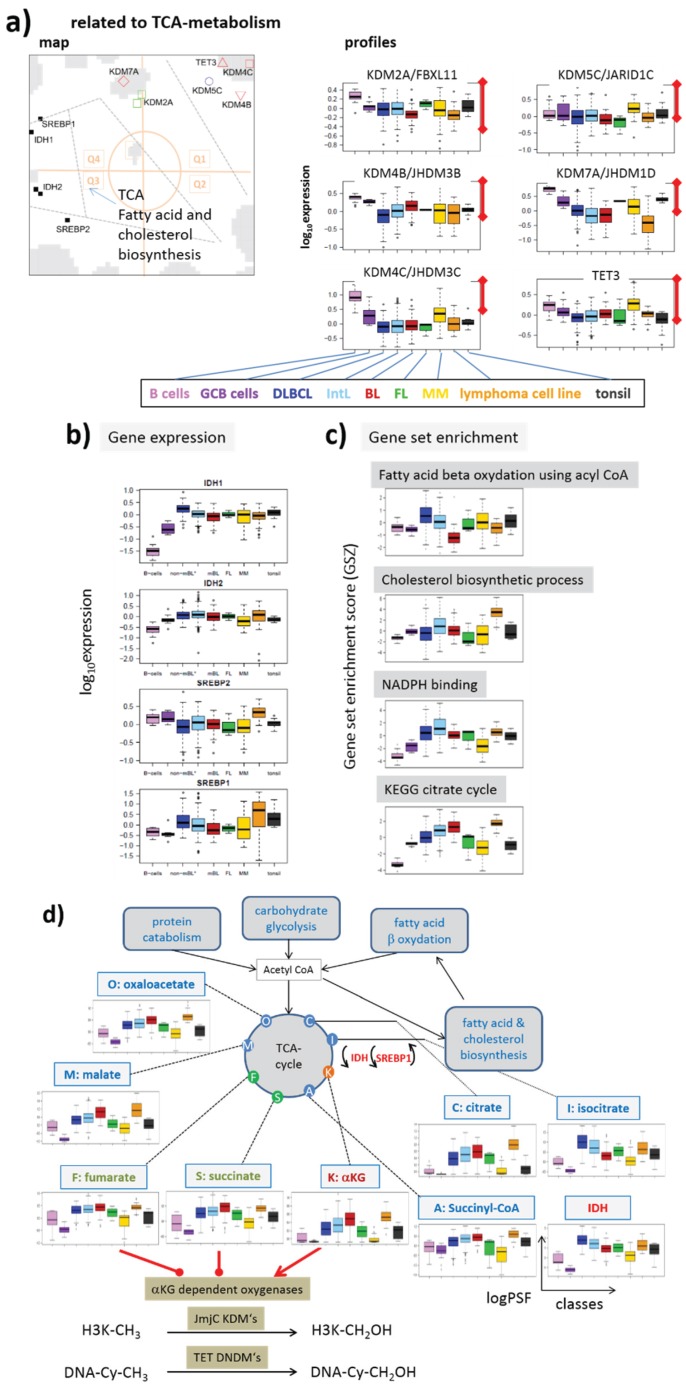
TCA-cycle related epigenetic compounds: (**a**) overview map and profiles of histone JmjC- and DNA TET-demethylases; (**b**) gene expression profiles of TCA-related enzymes and (**c**) gene set enrichment profiles of GO-gene sets related to TCA; (**d**) PSF-profiles of metabolic products along the TCA-cycle. Fumarate and succinate can act as antagonists of αKG to inhibit αKG-dependent dioxygenases. Note that the PSF profiles of all metabolites are very similar and virtually are anti-correlated with the expression of the demethylases shown in part a.

Genes coding enzymes of these families accumulate in Q1 in our lymphoma expression map indicating concerted down-regulation in lymphoma with maximum effects in DLBCL and IntL and partly also BL (Figure 9a). The expression of IDH1/2 genes governing α-KG activity shows strongly anti-correlated expression profiles as indicated also by their location in Q3 (Figure 9a,b, see also the correlation map in Figure 2c). Gene ontology (GO) gene sets related to TCA-cycle activity and particularly IDH1 expression show similar profiles reflecting strong activation in lymphoma and particularly in IntL, DLBCL and also BL (Figure 9d). The concerted activation of IDH1 and these processes is further supported by the co-expressed profiles of SREBP1/2, sterol regulated transcription factors linking mitochondrial NADPH-dependent IDH1 activity in the TCA cycle with lipogenesis and particularly cholesterol biosynthesis [88,89] (Figure 9b).

To estimate the activity of the intermediate products of the TCA cycle we applied PSF-analysis, which provides PSF profiles for these products. All these profiles are very similar showing strong alterations between healthy B cells and BCL on one hand and lymphoma on the other hand, but only subtle differences between the lymphoma subtypes (Figure 9d). This result reflects the almost “linear”, unbranched topology of the TCA cycle, where the activity of input compounds effectively passes through the whole cycle. In consequence, the PSF values of α-KG but also those of fumarate and of succinate are very similar resembling that of IDH1 and the GO gene sets of related processes. α-KG and fumarate/succinate are expected to concur in their inhibitory and activating effects on the oxygenase activities of the JmjC KDMs and TET-family DNDMs. The anti-correlated profiles of metabolites and enzymes suggest a net inhibitory effect between the energy metabolism and the activity of these epigenetic modifiers. The demethylases affected are erasers of H3K4me3 (KDM5C), H3K9me3 (KDM4B,C) and H3K27me3 (KDM7), and of DNA-CpG methylation marks (TET3). They are expected to induce activation of TrxG and PcG related expression programs and CpG hypermethylation, respectively (Figure 4c). A previous study shows that KDM4C is regulated by SREBP1 promoting this way the generation of succinate [38]. The latter two compounds indeed co-regulate with KDM4C in our data. Among the KDMs considered is also the H3K36me3 demethylating enzyme KDM2A, with possible consequences for DNA repair and/or transcriptional activity (see above).

### 3.5. Deregulation of Epigenetic Modifiers Governs Heterogeneity of Lymphoma

#### 3.5.1. Dysregulation of Epigenetic Writer-Eraser Equilibria Diminish Plasticity of B Cells during Maturation

**Figure 10:**
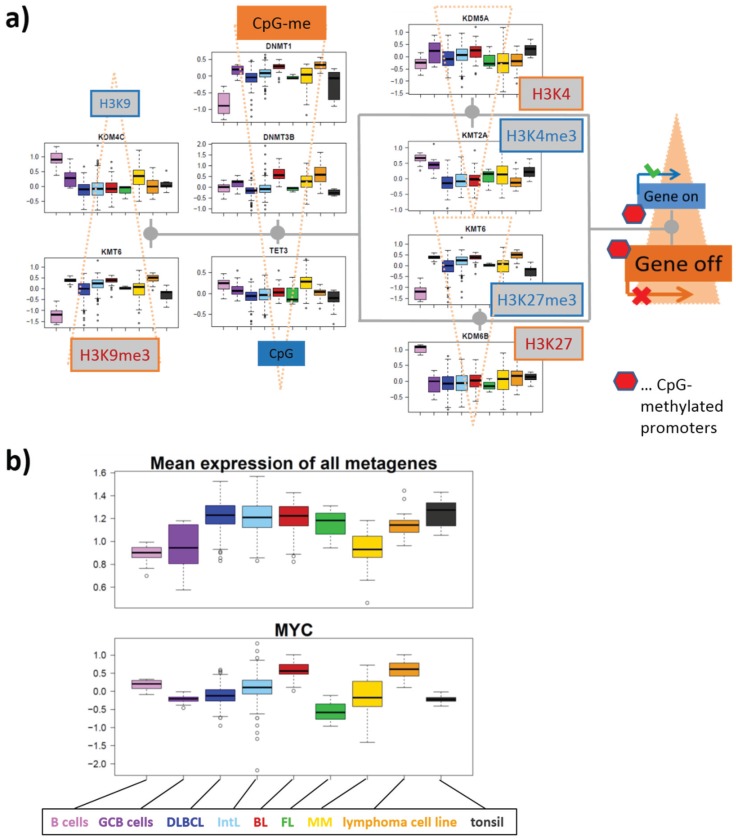
(**a**) The expression profiles of the methyltransferases and demethylases of H3K9, H3K4, H3K27 and of DNA-CpG’s suggest a shift of expression of the affected genes towards repressed and CpG-methylated promoter states. The scheme is redrawn from Figure 4 and supplemented by selected expression profiles of the respective enzymes determined from the lymphoma cohort studied. The triangles indicate the shift of the methylation-demethylation reactions in lymphoma compared with B cells deduced from the expression profiles from the respective enzymes. (**b**) Expression profiles of MYC and of the mean total expression averaged over all SOM-metagenes of each samples. Total expression is consistently activated in lymphoma except MM compared with B and GCB cells, whereas MYC is on high level in BL and IntL carrying genetic activating MYC defects.

In Figure 4 we discussed different scenarios of oncogenic perturbations in terms of a simplified scheme of epigenetic regulation. The systematic analysis of transcriptional activities of epigenetic modifiers presented in the previous subsections now enables us to compare the expected with the observed changes (Figure 10a). Compared with B cells, the equilibria of histone methylation reactions shift in direction of methylated H3K9 and H3K27 and demethylated H3K4 if one uses the expression data as a proxy for enzyme activities. These shifts suggest the increase of repressed and the decrease of active promoters in lymphoma accompanied by DNA hypermethylation, *i.e*., similar alterations as expected for EZH2 and MLL2 mutations (compare with the scenario in Figure 4b). Hence, the latter mutations and the expression changes of the enzymes suggest similar effects on DNA methylation and gene activities.

The diversification of lymphoma data into different subtypes and healthy controls enables a refined view, for example, on the changes of enzyme expression between different stages of B cell development in the germinal center. For some of the enzymes (e.g., KMT6/EZH2 and KDM6B/JMJD3) one finds similar expression levels in GCB cells and in part of the lymphoma subtypes: For example, EZH2 is silenced in resting B cells but massively up-regulated in GCB cells, which undergo rapid proliferation and immunoglobulin affinity maturation. JMJD3 shows nearly the opposite trend being highly active in B cells but nearly inactive in GCB cells and lymphoma. A similar, although less pronounced trend is found for KMD6A/UTX, another relevant K27DM [57].

These results reflect alterations of cellular programs during lymphocyte development, which are accompanied or even governed by epigenetic mechanisms. PRC2-mediated H3K27 trimethylation in healthy B cells is required on a selective core of PcG targets whose repression enables TF-dependent cell reprogramming [90], e.g., to transform naïve B cells into highly proliferative GCB cells. This reprogramming potentially also includes H3K4 methylation, which in concert with H3K27 methylation form bivalent promoter domains. These combined histone marks are required to poise genes for activation or deactivation in response to developmental and differentiation cues [91]. The resolution of the bivalent domains is mediated by the H3K4 and H3K27 demethylases. Mutations of MLL2 and EZH2 genes may both perturb this equilibrium. In consequence associated regulations go awry and lymphomas can ensue. Perturbations in the fine balance of GCB cell proliferation, differentiation and antigen exposure are assumed to lock GCB cells in an immature and proliferative state, which in collaboration with other lesions induce lymphoma.

Comparison of the transcriptional activities of the enzymes between the lymphoma subtypes reveals subtle differences, which suggest different types and degrees of disturbed equilibria. In all example profiles shown in Figure 10a one sees a monotonous increase of the mean enzyme expression from DLBCL over IntL to BL suggesting a continuous shift of the histone methylation equilibria. We recently presented indications for pronounced chromatin remodeling between BL and DLBCL affecting first of all transformations between repressed, poised and active promoter states [6]. These changes of promoter states potentially ensure alternative activation of proliferative (in BL and partly IntL) and inflammatory and developmental (in DLBCL and partly FL and IntL) expression programs and they are accompanied by aberrant DNA methylation in the promoter regions of the affected genes. Note that H3K4 and especially H3K27 methylation can tune not only developmental and “stemness” genes but also inflammatory processes needed to respond to external stimuli [58]. These different types of genes have in common that their function requires a high degree of plasticity for cell fate decisions. These decisions should induce different kinds of functional differentiation including maturation stages of the cells, their proliferative and metabolic activity and also the ability for adequate immune response.

#### 3.5.2. Activation of Gene Expression and of TCA Metabolism in Lymphoma Associates with Epigenetics

Bivalent and repressed promoters are prerequisites for the plasticity of the B cells required during their maturation in the GC. These genes can serve as hubs in TF networks that switch whole cascades of downstream genes either as suppressors, activators and/or enhancers of their transcriptional activity. In consequence, suppression of anti-proliferative programs and/or of activation of inflammatory processes is assumed to govern molecular mechanisms in lymphoma with respect to these functionalities. Increased proliferation requires up-regulation of the molecular machineries required for transcription and translation. In addition it needs activation of the metabolism delivering the energy needed for these processes as indeed observed in BL and IntL [10].

To judge this overall balance we calculated the mean total expression level of each sample using the metagene expression data obtained in our SOM analysis. The results clearly reveal a bimodal distribution with high expression levels in GC-derived lymphoma on one hand and with low expression levels in healthy B and GCB cells and in MM sharing similar expression signatures with B cells (Figure 10b). This result clearly supports the view that malignant transformations from B and/or GCB cells into GC-derived lymphoma are paralleled by the massive upregulation of the transcriptional activity in the cells. Interestingly, the profile of total expression partly resembles the PSF profiles of TCA metabolites (compare with Figure 9c) but anti-correlates with the expression profiles of genes located in spot I and particularly with that of KDM4C shown in Figure 7d. These results suggest that total gene expression in lymphoma and B cells is related to the TCA-energy metabolic activity, which, in turn, couples with the expression of epigenetic modifiers and particularly with KDM4C demethylating H3K9me3. Its low level in lymphoma (except MM) promotes trimethylation of H3K9 and recruitment of DNMTs which are on high level in lymphoma (see DNMT1, Figure 7b). In final consequence, one expects increased CpG-methylation in agreement with the scheme in Figure 10.

On the other hand, our data indicate subtle differences of the expression of a series of genes between B- and GCB-cells. Particularly, GCB-cells show higher total expression (Figure 10b) and higher activity of KEGG-TCA and NADPH related genes compared with B-cells (Figure 9c). This difference is possibly governed by a shift of the H3K9-methylation equilibrium, which suggests also changes in DNA promoter methylation (Figure 10a) of genes affecting the energy metabolism. We indeed identified differential methylation patterns between B- and GCB-cells, where increased methylation is found for PRC2-targets and repressed bivalent chromatin states in GCB-cells [6]. This result suggests that chromatin remodeling in the GC switches the state of metabolic activity between GCB- and B-cells.

We considered also another possible mechanism of global activation of transcription. Particularly, MYC can act as a universal amplifier of gene expression by hyper-activating still active genes via transcriptional pause release [92,93]. In consequence genes once activated by other mechanisms can be expected to become hyper-activated via aberrant MYC-overexpression. The expression profile of MYC (Figure 7b) however considerably differs from that of global expression. MYC is on high levels in BL and to a less degree also in IntL compared with DLBCL and FL, mainly due to genetic defects amplifying the MYC gene in BL and part of IntL. TCA metabolic activity better associates with the global transcriptional level in GC-derived lymphoma, suggesting mutual relations and possible consequences for epigenetics as discussed above.

#### 3.5.3. Asymmetric Activation of Methyl-Writers and -Erasers

**Figure 11:**
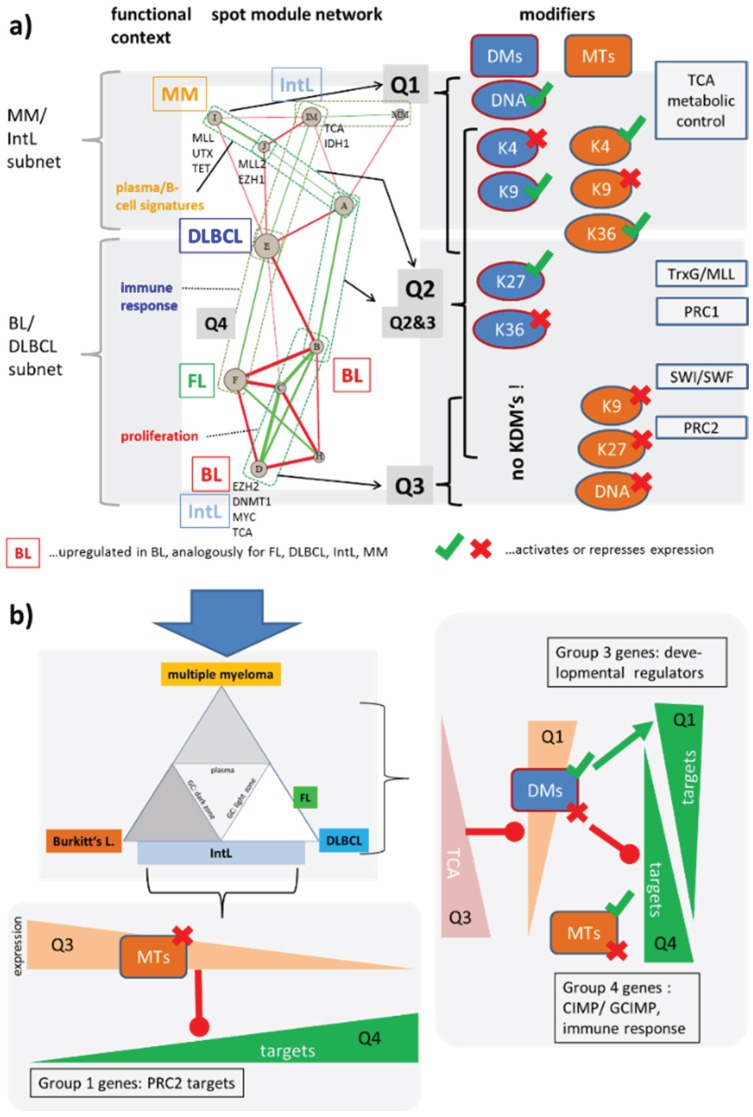
(**a**) Network of expression modules governing lymphoma heterogeneity. The nodes refer to the spot clusters extracted from the SOM analysis (for functional assignments see [6]). Green and red edges indicate positive and negative correlations with |w| > 0.3 using the weighted topological overlap correlation measure (see also Figure 2). A “backbone” of correlated modules can be assigned to the Q1–Q3 quadrants of the SOM used to map enzyme activities. Accumulation of different types of modifiers and complexes in Q1–Q3 is shown in the right part. Q4 is almost depleted from modifiers. A detailed list of the modifiers found in each spot is given in Figure S3. The network can be roughly divided into two subnets as described in the text. (**b**) The DLBCL/BL and MM/IntL subnets explain the expression changes of three different groups of genes identified in [6] (see text).

Our study clearly shows that the expression of nearly all enzymes considered alters markedly between the lymphoma subtypes. For a holistic view we make use of the fact that SOM cartography maps the genes in an organized way. The structure of the map provides information about the underlying regulatory net because the arrangement of spots reflects their mutual co-variance structure (Figure 2c). We assigned the location of the epigenetic modifiers in the map to the respective spots (see Table 1), and with a more coarse resolution to the quadrants Q1 to Q4. Interestingly, we found strong depletion of epigenetic modifiers in Q4 opposed by their enrichment in Q2 and particularly also in Q1 and Q3 (Figure 6). In the next step we rearranged the network of expression modules to better resolve its covariance structure (Figure 11). It clearly reveals a “backbone” of mutually correlated modules, which sequentially connects spots from Q1 to Q3. A second backbone is formed by correlated spots mostly located in Q4 and partly in Z (spot MM) and Q3 (IM). It forms an almost separated entity connected via anti-correlated edges (in red) from the first, main backbone. The spots and thus also the respective quadrants contain co-regulated genes specifically up-regulated in different subtypes as indicated in Figure 11. Importantly, almost each of the regulatory modes also affects a group of epigenetic modifiers. In other words, (de)regulation of epigenetics covers the whole transcriptional landscape of lymphoma. Moreover, the epigenetic modifiers enrich within the spot clusters when compared with the total number of genes in the spots (Fishers exact test: *p* = 3.7 × 10^−6^). Hence, epigenetic modifiers are affected by (de-)regulatory effects with higher probability than expected by chance.

The network can be decomposed into a subnet, which mainly refers to genes that antagonistically switch between BL on one hand and DLBCL/FL on the other hand. Interestingly, this subnet accumulates methyltransferases in Q3 that tend to repress gene expression of their target genes leading to antagonistic expression profiles in Q4 (the detailed assignment of enzymes to each of the spots is given in Supplementary Figure S3). The imbalance between the gene expression of methyltransferases and demethylases between Q3 and Q4 can be rationalized partly by the requirement of maintenance methylation of DNA-CpG and histone methylation marks after cell division and DNA replication, which requires high activities of methyltransferases. The question whether upregulation of KMT and DNDMT expression in the highly proliferative subtypes BL and partly IntL ensures maintenance of DNA and histone methylation patterns or whether it leads to progressive loss of methylation requires further studies.

Figure 11b illustrates this antagonism between BL and DLBCL using a triangular scheme of lymphocyte development and lymphoma heterogeneity. Particularly the K27MTs in Q3 are expected to inhibit PRC2 targets, which indeed accumulate in the anticorrelated region Q4. These genes were subsumed as group 1 genes in [6], being hypermethylated and overexpressed in lymphoma compared with the controls. In addition, compounds of the PRC1 and SWI/SWF complexes co-regulate with these MTs and their targets. This parallel suggests that the stabilization of repressed promoters and the opening/closing of chromatin are mechanisms that change the expression patterns between BL and DLBCL.

Another subnet in Figure 11a contains genes that switch between the MM and IntL. It accumulates methyltransferases and demethylases in Q1 that repress or activate expression. Most of the demethylases are JmjC-family enzymes, which are repressed by TCA products such as fumarate and succinate as illustrated in the right panel of Figure 11b. These demethylases together with the KMTs in Q1 then either repress or activate expression of their targets giving rise to group 3 and group 4 genes as genes that antagonistically change their expression between Q1 and Q4 [6]. These gene groups are enriched in developmental regulators, genes related to immune response, PRC2 targets and also CIMP/GCIMP genes hypermethylated in colon and brain cancer, respectively.

Both subnets overlap in Q2 collecting most of the epigenetic modifiers including activating and repressing ones without clear preference (Figure 11a). This overlap region contains modules that switch expression between (GC)B cells and lymphoma. We hypothesize that the underlying modes regulate transcriptional programs differentiating between healthy B and GCB cells. Other enzymes localize near spots H and MM referring to early and late stages of B cell maturation, respectively [12].

In summary, network analysis of the epigenetic modifiers identifies two subnets related to differential expression between BL and DLBCL, and between MM and IntL subtypes, respectively. The former subnet is governed by methyltransferases upregulated in BL and repressing transcription of their target genes. The latter one contains demethylases which are presumably under metabolic control and which can activate and/or repress their targets.

## 4. Conclusions

Lymphomas show a very diverse pattern of transcriptional activity of histone and DNA methylating and demethylating enzymes and of associated reader complexes. Basic epigenetic functions in healthy B cells seem to ensure a high level of plasticity for cell fate decisions between biological functions, such as proliferation, immune response and differentiation that sequentially switch on and off during B cell maturation in the germinal center. Repressed and poised promoter states of key regulatory genes seem to play a pivotal role in this process. The fine balance between histone modifications activating or repressing transcription is governed by methylation equilibria of lysine histone side chains and of DNA CpGs as well.

In lymphomas this balance becomes disturbed in a subtype specific fashion leading to de-regulations of functional programs, which, in final consequence, induce lymphomagenesis. Driver mutations directly affecting epigenetic modifiers, such as EZH2 and MLL2, represent one type of initial events causing malignant transformations in lymphocytes [36]. Another option can be seen in the massive upregulation of the energy metabolism in the cell and metabolic coupling with epigenetics, where metabolites act as cofactors of JmjC-type demethylases. Finally, also indirect effects, e.g., if disturbed gene regulations affect epigenetic modifiers with downstream consequences for the epigenome.

The main result of our systematic study is the finding that the expression levels of nearly all fifty enzymes studied markedly change between the sample-classes considered. Lymphoma biology apparently associates with deregulation of large parts of the epigenetic machinery of the cell. Preliminary results on enzymes affecting histone marks other than methyl groups, such as acetyl groups, support this view. Hence, understanding of epigenetic deregulation in lymphoma must go beyond simple schemes using only a few modes of regulation. We showed that the systematic “cartography” of epigenetic modifiers onto the expression landscape of a disease using SOM machine learning as the basic technique enables a holistic view on the heterogeneity of (de)regulation by epigenetic modifiers. A comprehensive, data driven network analysis provided indications that (de-) regulation of epigenetic enzymes is associated with virtually all modes of transcriptional regulation identified in lymphoma.

On the other hand, our network analysis showed that BL and DLBCL differ by the imbalance of repressive and poised promoters, which is governed first of all by methyltransferases and to a less degree by demethylases. The underrepresentation of demethylases in this regulation has the interesting consequence that in DLBCL only a small amount of modifying enzymes become upregulated, whereas BL is characterized by massive activation of modifiers. Another interesting finding suggests that coupling of epigenetics with the metabolic activity of the cell can be an important factor in lymphomagenesis, even if mutations in genes affecting metabolic pathways are missing. The question is whether metabolic coupling is a general phenomenon in cancer that generates characteristic DNA methylation patterns in the genome of B cells resembling CIMP and/or GCIMP phenotypes in colon cancer and glioma, respectively.

A series of other open questions directly ensues from our study: (i) The role of the enzymes should be estimated based on their chemical activities and not only on their expression levels. (ii) Molecular networks of epigenetic regulation beyond the simple scheme discussed here have to be established and studied using experimental and also theoretical modeling approaches. (iii) The further study of the coupling between the TCA-metabolism and epigenetics suggested here requires the direct measurement of metabolites. (iv) Meta-analyses including different cancer entities are required to identify more ubiquitous and more specific modes of epigenetic regulation. (v) Initiating epigenetic “driver”-effects have to be distinguished from downstream “passenger”-effects (“Who is the driver?”). In addition, methods have to be developed that allow this differentiation. (vi) The understanding of molecular mechanisms of cancer requires integrative analysis of omics data including, e.g., gene expression, mutations, DNA methylation and chromatin states. (vii) Interpretation of data on B cell lymphoma requires a systems biology view for better understanding of epigenetic effects, including regulatory modes governing maturation of healthy B-cells. (viii) The number of enzymes considered has to be extended beyond methylation to include also other histone modifications, such as acetylation, ubiquitylation and others, for which one can expect also massive deregulation effects in lymphoma.

## Supplementary Files

Supplementary File 1
