# Supplementary material for: Epigenetic Heterogeneity of B-Cell Lymphoma: Chromatin Modifiers
Source: Genes (Basel). 2015 Oct 21;6(4):1076–112. doi: 10.3390/genes6041076 (PMC4690029; doi:10.3390/genes6041076)
Supplement: Supplementary File 1 [file genes-06-01076-s001.pdf]

Supplementary Figures

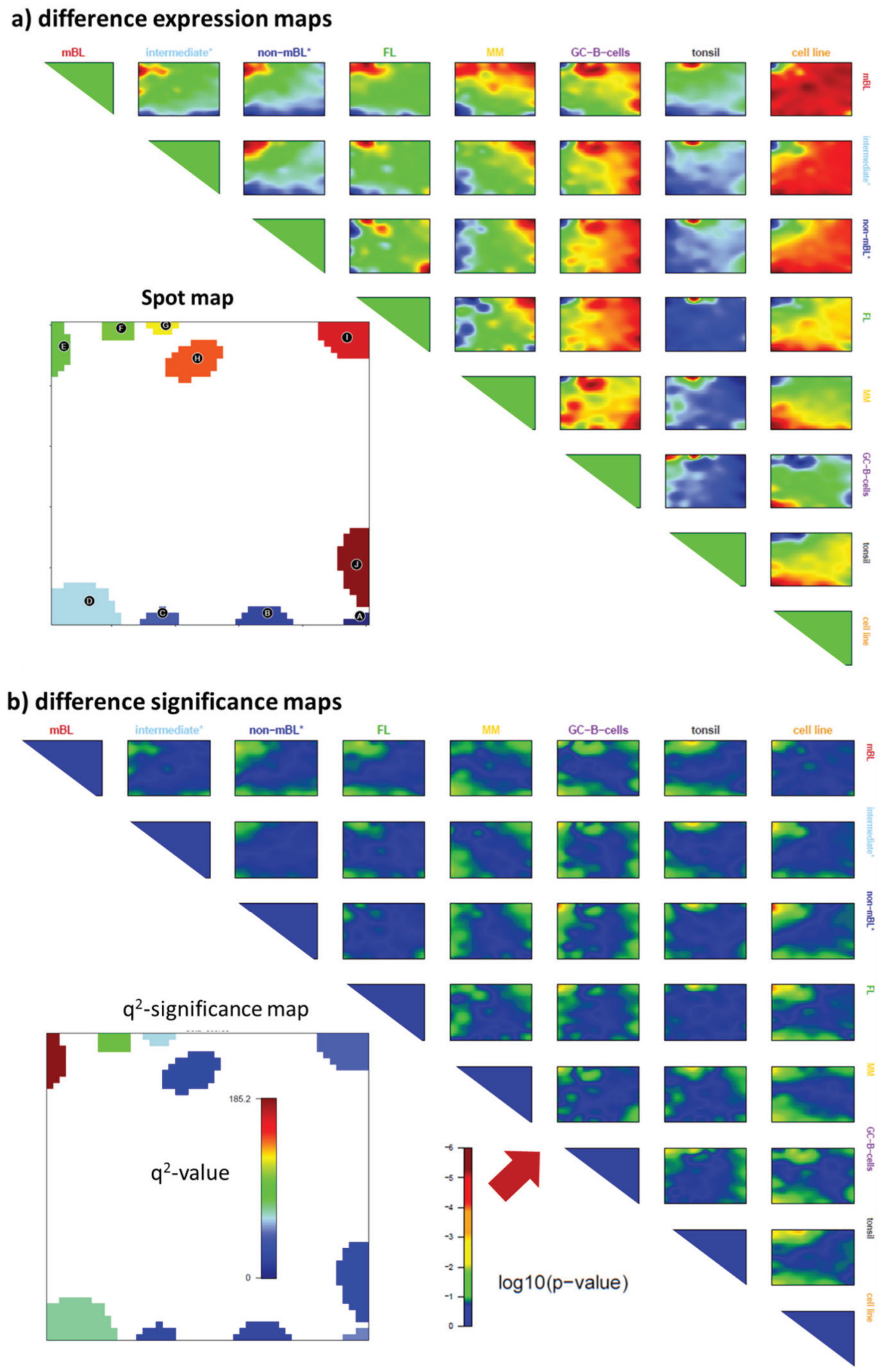

Figure S1. Maps of differential expression and of significance. The small maps visualize differential expression between all pairwise combinations of classes (**a**) and the respective significance maps (**b**) which color code the log ***p***-values extracted from a shrinked *t*-test [1]. Differential expression was calculated separately for each pixel of the SOM. The large maps assign the spots (a) and significance level of co-expression of the genes in each of the spots as estimated using a multitest adjusted q^2-correlation test (b, [2]). The maps enable us to identify regions of significant differential expression for each pairwise combination of classes. One sees that spots and associated genes show significant differential expression between selected classes, especially in the left (Q3 and Q4: spots D–F) and the lower (Q2: spots A, B, and J) part of the map.

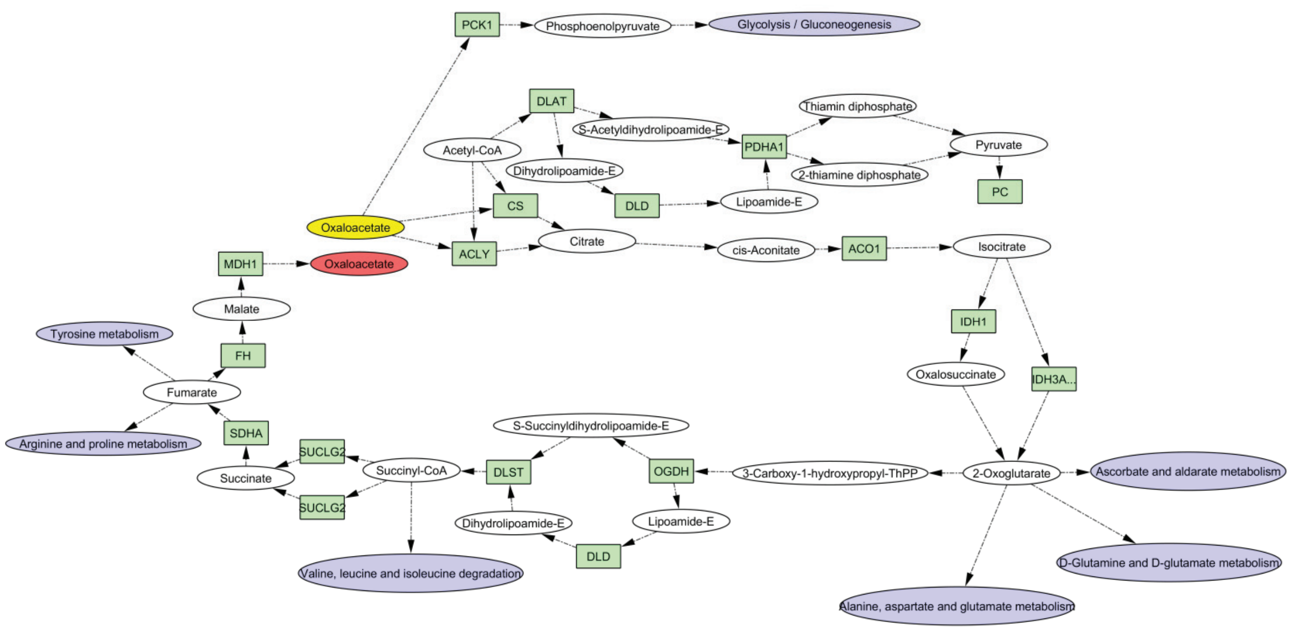

Figure S2. PSF analysis of the TCA pathway. The canonical KEGG TCA pathway was converted into a linear one-directional form. Green rectangles represent enzymes, white circles are compounds, and violet nodes are target processes. The 'Oxaloacetate' node is drawn in two places: the yellow circle represents the pathway input and the red circle represents the final output.

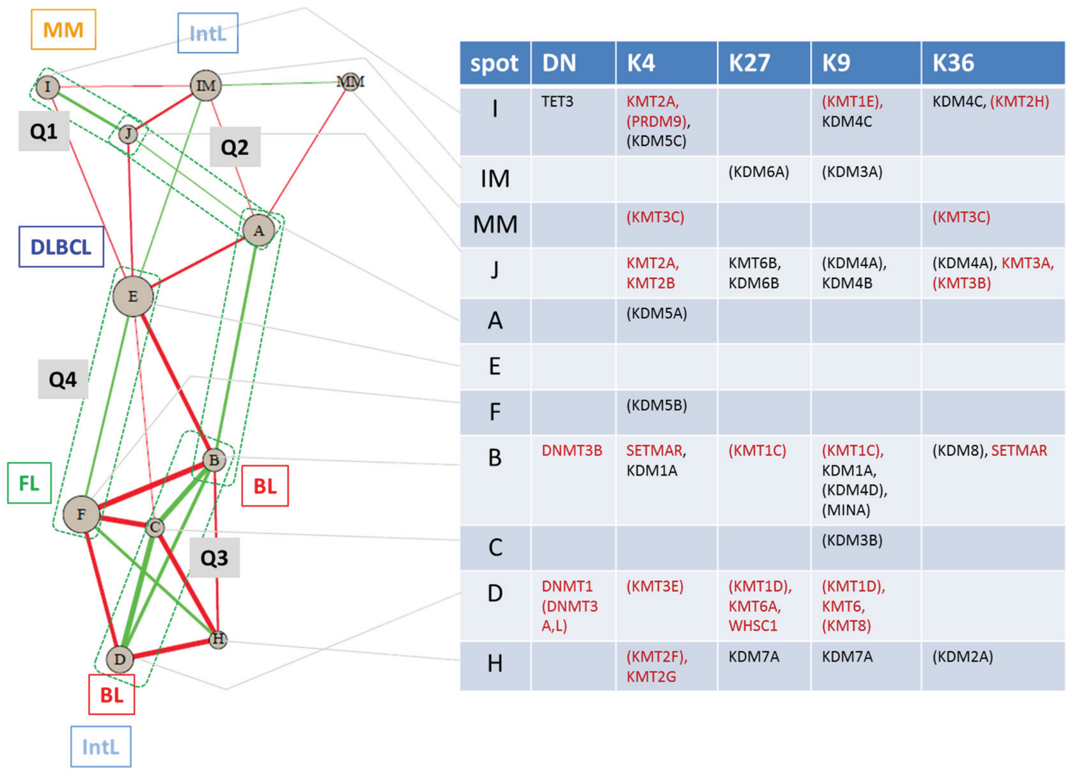

spot	DN	K4	K27	K9	K36
I	TET3	KMT2A, (PRDM9), (KDM5C)		(KMT1E), KDM4C	KDM4C, (KMT2H)
IM			(KDM6A)	(KDM3A)	
MM		(KMT3C)			(KMT3C)
J		KMT2A, KMT2B	KMT6B, KDM6B	(KDM4A), KDM4B	(KDM4A), KMT3A, (KMT3B)
A		(KDM5A)			
E					
F		(KDM5B)			
B	DNMT3B	SETMAR, KDM1A	(KMT1C)	(KMT1C), KDM1A, (KDM4D), (MINA)	(KDM8), SETMAR
C				(KDM3B)	
D	DNMT1 (DNMT3 A,L)	(KMT3E)	(KMT1D), KMT6A, WHSC1	(KMT1D), KMT6, (KMT8)	
H		(KMT2F), KMT2G	KDM7A	KDM7A	(KDM2A)

Figure S3. Network of expression modules governing lymphoma heterogeneity. The table in the right part lists the methyltransferases (red letters) and demethylases (black letters) located within (or next to) each of the spots.

References

1. Wirth, H.; von Bergen, M.; Binder, H. Mining SOM expression portraits: Feature selection and integrating concepts of molecular function. *BioData Mining* **2012**, doi:10.1186/1756-0381-5-18.
2. Binder, H.; Wirth, H.; Arakelyan, A.; Lembcke, K.; Tiys, E.S.; Ivanishenko, V.; Kolchanov, N.A.; Kononikhin, A.; Pop, I.; Nikolaev, E.N.; *et al*. Time-course human urine proteomics in space-flight simulation experiments. *BMC Genomics* **2014**, doi:10.1186/1471-2164-15-S12-S2.

© 2015 by the authors; licensee MDPI, Basel, Switzerland. This article is an open access article distributed under the terms and conditions of the Creative Commons Attribution license (http://creativecommons.org/licenses/by/4.0/).
